# Genome-wide identification and expression analysis of carotenoid cleavage oxygenase genes in Litchi (*Litchi chinensis* Sonn.)

**DOI:** 10.1186/s12870-022-03772-w

**Published:** 2022-08-09

**Authors:** Xiao-Qi Yue, Yue Zhang, Cheng-Kun Yang, Jian-Guo Li, Xia Rui, Feng Ding, Fu-Chu Hu, Xiang-He Wang, Wu-Qiang Ma, Kai-Bing Zhou

**Affiliations:** 1 Engineering Research Center of Selecting and Breeding New Tropical Crops Varieties, Ministry of Education, Horticulture College, Hainan University, Hainan 570311 Haikou, China; 2 Key Laboratory for Quality Regulation of Tropical Horticultural Crops of Hainan Province, Horticulture College, Hainan University, Hainan, 570311 Haikou, China; 3 Guangdong Litchi Engineering Research Center, College of Horticulture, South China Agricultural University, Guangzhou, 510642 China; 4 Guangxi Crop Genetic Improvement and Biotechnology Key Laboratory, Guangxi Academy of Agricultural Sciences, Nanning, 530007 Guangxi China; 5 Key Laboratory of Tropical Fruit Tree Biology of Hainan Province, Hainan Academy of Agricultural Science, Haikou, 571100 China

**Keywords:** Litchi, *CCO* genes, Expression analysis, Flower control, Fruit development and maturation, Postharvest storage

## Abstract

**Background:**

Carotenoid cleavage oxygenases (CCOs) include the carotenoid cleavage dioxygenase (CCD) and 9-cis-epoxycarotenoid (NCED), which can catalize carotenoid to form various apocarotenoids and their derivatives, has been found that play important role in the plant world. But little information of *CCO* gene family has been reported in litchi (*Litchi chinensis* Sonn.) till date.

**Results:**

In this study, a total of 15 *LcCCO* genes in litchi were identified based on genome wide lever. Phylogeny analysis showed that *LcCCO* genes could be classified into six subfamilies (CCD1, CCD4, CCD7, CCD8, CCD-like, and NCED), which gene structure, domain and motifs exhibited similar distribution patterns in the same subfamilies. MiRNA target site prediction found that there were 32 miRNA target sites in 13 (86.7%) *LcCCO* genes. *Cis*-elements analysis showed that the largest groups of elements were light response related, following was plant hormones, stress and plant development related. Expression pattern analysis revealed that *LcCCD4*, *LcNCED1,* and *LcNCED2* might be involving with peel coloration, *LcCCDlike-b* might be an important factor deciding fruit flavor, *LcNCED2 and LcNCED3* might be related to flower control, *LcNCED1* and *LcNCED2* might function in fruitlet abscission, *LcCCD4a1*, *LcCCD4a2, LcCCD1, LcCCD4, LcNCED1, and LcNCED2* might participate in postharvest storage of litchi.

**Conclusion:**

Herein, Genome-wide analysis of the *LcCCO* genes was conducted in litchi to investigate their structure features and potential functions. These valuable and expectable information of *LcCCO* genes supplying in this study will offer further more possibility to promote quality improvement and breeding of litchi and further function investigation of this gene family in plant.

**Supplementary Information:**

The online version contains supplementary material available at 10.1186/s12870-022-03772-w.

## Background

Carotenoids are isoprenoid-based compounds, also named as a kind of important natural pigments. Carotenoids can be found from archaea and eubacteria to eukaryotes (like animals, higher plants, fungi and algae), play vital roles in photosynthesis, signaling, antioxidant properties, electron transport, and light absorption [1–3]. Carotenoid cleavage oxygenase (CCO) is a type of important enzyme in the carotenoid metabolic pathway, which can catalyze carotenoid to various apocarotenoids and their derivatives to perform important biological functions in plants. The CCOs can be divided into two forms, one named as CCD (Carotenoid Cleavage Dioxygenase) and the other is NCED (9-cis-epoxycarotenoid dioxygenase) depending on whether the substrates are epoxidated [4].

The *CCO* gene family has been reported commonly involved in the formation of flavor and scent, coloration, even growth and development, ecological adaptation in plants through regulating the carotenoids pathway. In *Arabidopsis thaliana*, *AtCCO* genes family includes 4 *CCD* and 5 *NECD* genes [5]. At the beginning of the previous work, the *CCO* genes were divided into five categories, which included *CCD1*, *CCD4*, *CCD7*, *CCD8*, and *NCED* [6, 7]. Recently, another category of *CCO* genes called *CCD*-like (*CCDL*) was found in grape (*Vitis vinifera)*, tomato (*Solanum lycopersicum*), apple (*Malus* × *domestica*) and Sugar cane (*Saccharum*) [8–11]. In general, different categories of *CCO* genes exhibit different roles. CCD1 can catalyze carotenoids into several metabolites like α-ionone, β-ionone, and geranylacetone, which play an important role in the formation of the flavor and scent of horticultural plants [12, 13]. *CmCCD4a* gene contributes to white color formation in chrysanthemum petalsonly (*Chrysanthemum morifolium* Ramat.) by catalyzing the carotenoids to colorless compound [14]. *GmCCD4* in soybeans was also found to be a negative regulator of carotenoid content [15]*.* Natural Variation in *CCD4* gene promoter is a major genetic determinant of natural variation in C30 apocarotenoids which is responsible for red coloration of citrus peel [16]. The CCD7 and CCD8 enzymes are involved in the biosynthesis of the strigolactone (a relatively novel apocarotenoid hormone), which could control shoot branching and reproductive development and regulate plant responses to drought and salt stress [7, 17–19]. NCED is the key enzyme for the biosynthesis of abscisic acid (ABA), which is closely involved in the fruit development, ripening and senescence. Such as *FaNCED1* RNAi in strawberry (*Fragaria* × *ananassa*) fruits could decline the ABA content significantly and resulted in uncolored fruits [20]. The application of exogenous ABA could accelerate the accumulation of anthocyanin by increasing the expression of *NCED* genes to promote the coloration of strawberry [21], grape [22], sweet cherry [23] and litchi fruits [24]. Some reports showed that ABA might be correlate with the fruit abscission of the citrus, apple and litchi [44–46]. The ABA content increased by uniconazole spraying was helpful to the flower control and fruit retention of litchi [48, 49]. ABA was considered to play key role during the fruit senescence, which was the most important factor that deciding the shelf life of fruit [53, 54]. Additionally, ABA is also reported to be related to the bud dormancy, leaf abscission, and responses to diverse environmental stresses [25]. Together, these studies showed that *CCO* genes play an important role in plant world.

Litchi (*Litchi chinensis* Sonn.) is a member of the sapindaceae family and an important subtropical and tropical economic fruit which is famous by its attractive skin colour and exotic flavour. But there are still some challenges in the litchi planting industry, such as the peel coloration (‘stay green’ or pigmenting uneven problem in some varities like ‘Feizixiao’), fruit abscission, flowering control and postharvest preservation. The *CCO* gene family have been reported to be involved in important biological functions in the plants [1–3]. However, this gene family has not been identified in litchi. In this study, genome-wide identification of *CCO* gene family had been conducted, and their gene structure, domain, motif, phylogenetic relationship, miRNA target sites, *cis*-elements, 3D protein structure, and expression patterns were comprehensively analyzed. The study may provide a solid foundation for future functional studies of *CCO* genes in litchi and other fruit trees.

## Results

### Identification of *LcCCO* genes and their physicochemical properties

After homology search, a total of 15 *LcCCO* genes were identified in litchi. Physicochemical properties analysis found that the largest protein was LcCCD1, which contained 1434 amino acids, the smallest protein was LcCCD4c1, which contained 303 amino acids. MW of LcCCO proteins ranged from 16.25 kDa to 34.23 kDa, pI ranged from 5.54 to 8.15. Instability index analysis revealed that LcCCD1, LcCCDlike-a, LcCCDlike-b, LcCCD4c1, LcCCD4c2, and LcCCD8b were stable proteins(Instability index < 40), and the rest were unstable proteins. Aliphatic index of LcCCO proteins ranged from 75.61 to 84.62. Grand Average of Hydropathicity analysis showed that all LcCCO proteins were hydrophilic protein. Subcellular localization prediction exhibited that most of LcCCO proteins (11, 73.3%) were located in chloroplast, three LcCCO proteins (LcCCDlike-a, LcCCDlike-b, and LcCCD4c1) were located in cytoplasm, one LcCCO proteins (LcCCD8b) was located in mitochondrion (Table 1).

**Table 1:** Basic information of *LcCCO* gene family

***LcCOO* genes**	**Gene ID** **in genome**	**Genomic** **position**	**Number** **of amino** **Acids** **(aa)**	**MW (KDa**)	**pI**	**Instability index**	**Aliphatic index**	**Grand** **average of** **hydropathicity**	**Subcellular** **localization** **prediction**
*LcCCD1*	*LITCHI004397.m1*	Chr14:923,933–940,844	1434	162.43	6.12	35.41	84.62	-0.231	Chloroplast
*LcCCDlike-a*	*LITCHI017175.m1*	Chr1:43,820,531–43,837,433	1172	131.99	5.54	36.52	75.61	-0.314	Cytoplasm
*LcCCDlike-b*	*LITCHI001770.m1*	Chr5:30,309,771–30,311,628	359	40.68	5.90	29.50	80.56	-0.179	Cytoplasm
*LcCCD4*	*LITCHI017848.m1*	Chr15:133,065–136,343	589	64.70	6.65	42.13	80.44	-0.202	Chloroplast
*LcCCD4a1*	*LITCHI000409.m1*	Chr5:11,437,371–11,439,125	584	65.72	7.23	49.31	80.77	-0.142	Chloroplast
*LcCCD4a2*	*LITCHI000415.m1*	Chr5;11,580,107–11,582,498	584	65.70	7.23	48.84	82.11	-0.099	Chloroplast
*LcCCD4b*	*LITCHI000422.m1*	Chr5;11,719,278–11,721,297	577	64.97	8.15	46.41	81.56	-0.182	Chloroplast
*LcCCD4c1*	*LITCHI015832.m1*	Chr1;19,365,348–19,366,692	303	34.23	6.37	39.62	80.03	-0.230	Cytoplasm
*LcCCD4c2*	*LITCHI015831.m1*	Chr1;19,334,658–19,337,656	566	62.63	6.48	31.10	81.64	-0.277	Chloroplast
*LcCCD7*	*LITCHI006417.m1*	Chr11;1,151,592–1,157,255	628	70.57	7.27	49.75	80.51	-0.269	Chloroplast
*LcCCD8a*	*LITCHI006516.m1*	Chr11;1,903,352–1,906,489	548	61.54	6.28	42.19	76.17	-0.426	Chloroplast
*LcCCD8b*	*LITCHI017183.m1*	Chr1;43,942,769–43,947,076	566	62.63	6.48	31.10	81.64	-0.277	Mitochondrion
*LcNCED1*	*LITCHI012579.m1*	Chr2;12,609,603–12,614,092	596	66.73	6.82	47.43	77.03	-0.389	Chloroplast
*LcNCED2*	*LITCHI028785.m1*	Chr9;17,432,409–17,435,810	598	66.85	6.51	43.21	80.87	-0.300	Chloroplast
*LcNCED3*	*LITCHI015114.m1*	Chr1;8,869,747–8,872,765	601	67.21	6.95	40.23	82.63	-0.331	Chloroplast

### Phylogenetic analysis

**Fig. 1:**
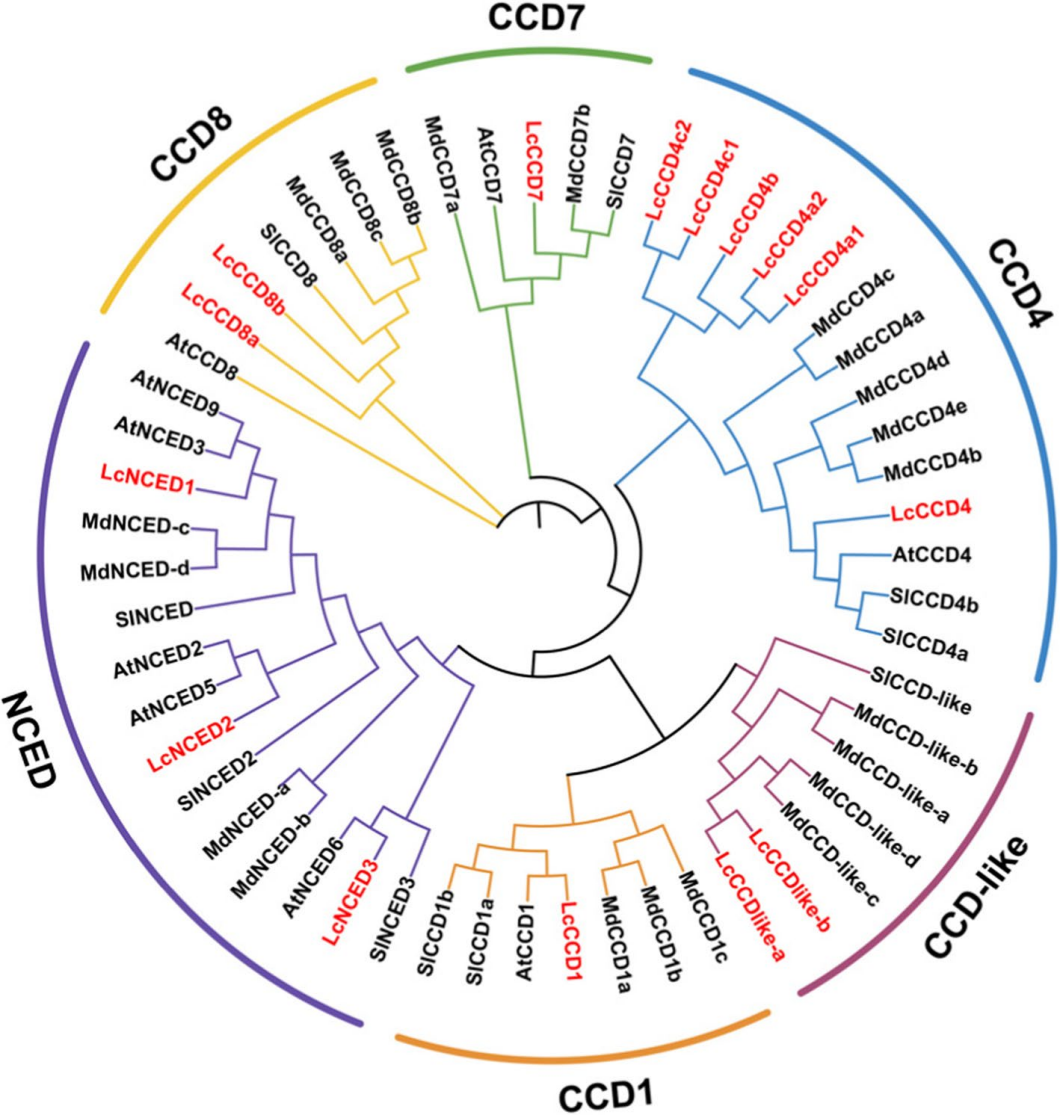
Phylogenetic tree was constructed by ML method. Red character represented CCO proteins from *Litchi chinensis* Sonn., black character represented CCO proteins from *Arabidopsis thaliana*, *Solanum lycopersicum*, *Malus* × *domestica*

To better understand the evolutionary relationships among CCD proteins, maximum-likelihood (ML) methods were adopted to construct an unrooted phylogenetic tree which containing 55 CCO proteins from the following four species: *Arabidopsis thaliana* (9), *Solanum lycopersicum* (10), *Malus* × *domestica* (21) and *Litchi chinensis* Sonn (15) (Fig. 1). The result showed that the 55 CCD proteins could be divided into six subfamilies (CCD1, CCD4, CCD7, CCD*8*, CCD-Like, and NCED) (Fig. 1). CCD4 are the largest subfamily, including six members (*LcCCD4*, LcCCD4a1, LcCCD4a2, LcCCD4b, LcCCD4c1, and LcCCD4c2), while CCD7 is the smallest subfamily, just one member (LcCCD7). Compared to *Arabidopsis thaliana*, the number of *LcCCO* genes is about twice as much as the two formers.

### Gene structure, domain, motif and chromosomal arrangement analysis

**Fig. 2:**
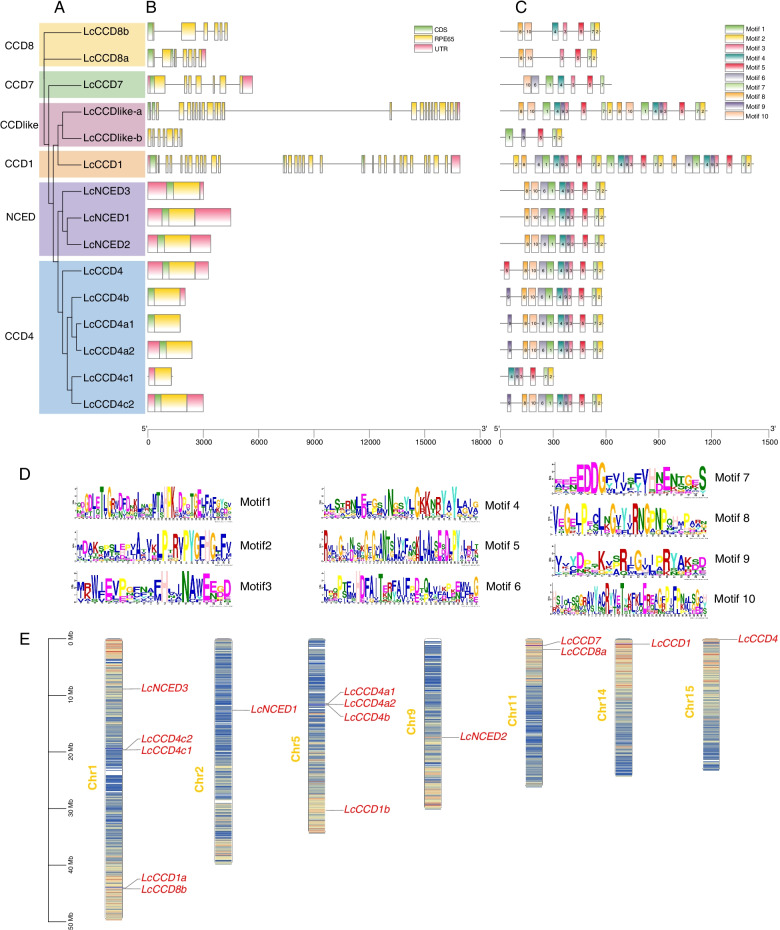
Gene structure, conserved domain and motif of *LcCCO* genes. **A** Phylogenetic tree of *LcCCO* genes. **B** The distribution of gene structure and conserved domain. **C** The distribution of conserved motif, **D** motif elements. **E** Chromosomal distribution of *LcCCO* genes

Gene structure analysis showed that the numbers of exon of *LcCCO* genes ranged from 1 to 32. *CCD1* subfamily contained the largest number of exons, following were the *CCD7* and *CCD8* subfamily, *CCD4* and *NCED* subfamily just had one exon. *LcCCO* genes in the same subfamilies displayed similar structure distribution patterns (Fig. 2B). Conserved domain analysis exhibited that all of *LcCCO* genes contained a RPE65 domain (Fig. 2B). Motif analysis showed that all of *LcCCO* genes had the motif 5 and motif 7, and like conserved domain, displayed similar patterns in the same subfamilies. Such as in *NCED* family, the distribution pattern of motifs of each member began with motif9, 10, 6, 8, 10, 6, 1, 4, 9, 3, 5, 7, and 2 from N-terminal to C-terminal (Fig. 2C-D). Chromosomal distribution analysis showed that *LcCCO* genes located in seven chromosomes (Fig. 2E). Chromosomes1 and 5 (Chr1 and Chr5) contained nine (60%) *LcCCO* genes (five and four respectively). Chr11 had two (13.3%) *LcCCO* genes. Chr2, 9, 14 and 5 carried only one (6.7%) *LcCCO* gene. *LcCCDlike-b*, *LcNCED2,* and *LcCCD4* were located in the regions with high gene density.

### Prediction of miRNA target site of *LcCCO* genes

MiRNA target site prediction showed that a total of 31 miRNA target sites could be found in 13 (86.7%) *LcCCO* genes with the exception of *LcCCDlike-b* and *LcNCED3* (Table 2). Among all *LcCCO* genes, *LcCCD1* and *LcCCDlike-a* existed the most miRNA target sites, which could be targeted by 10 and 7 miRNAs separately. *LcCCD4c1* and *LcCCD7* just had one miRNA target site. In the same subfamily, we found that some members could be targeted by a same miRNA. Such as *LcCCD4a1, LcCCD4a2, LcCCD4b,* and *LcCCD4c2,* which belonged to the *CCD4* subfamily, could by targeted by Lc-miRN23 simultaneously. *LcCCD1* and *LcCCDlike-a*, which belonged to *CCD1* subfamily, could be targeted by Lc-miRN58 concurrently, but the *LcCCDlike-a* existed two different Lc-miRN58 target sites. More generally, *LcCCO* genes in the same or different subfamilies were targeted by different miRNAs. Such as *LcNCED1*, *LcNCED2,* and *LcNCED3*, which belonged to *NCED* subfamily, there were no common miRNA targets.

**Table 2:** The potential miRNA target sites of *LcCCO* genes

miRNA	Target	Expectation	miRNA Length	Target_start	Target end	Inhibition	Multiplicity
Lc-miR408b/d/f	*LcCCD1*	3.5	20	2577	2597	Cleavage	1
Lc-miR172h	*LcCCD1*	4	20	2445	2465	Cleavage	1
Lc-miR408a/c/e	*LcCCD1*	4.5	20	2578	2598	Cleavage	1
Lc-miR160c/d	*LcCCD1*	5	20	2057	2077	Cleavage	1
Lc-miR172a/b/c/d/e/i/j	*LcCCD1*	5	20	2445	2465	Cleavage	1
Lc-miRN19	*LcCCD1*	5	20	3794	3814	Cleavage	1
Lc-miRN58	*LcCCD1*	5	21	1942	1963	Cleavage	1
Lc-miRN49	*LcCCDlike-a*	4	21	2941	2962	Cleavage	2
Lc-miRN49	*LcCCDlike-a*	4.5	21	1267	1288	Cleavage	2
Lc-miR156g/l	*LcCCDlike-a*	5	20	80	101	Cleavage	1
Lc-miR397a/b	*LcCCDlike-a*	5	20	1752	1772	Cleavage	2
Lc-miR397a/b	*LcCCDlike-a*	5	20	3351	3371	Cleavage	2
Lc-miR397c/d	*LcCCDlike-a*	5	19	1753	1772	Cleavage	2
Lc-miR397c/d	*LcCCDlike-a*	5	19	3352	3371	Cleavage	2
Lc-miRN24	*LcCCDlike-a*	5	20	323	343	Cleavage	1
Lc-miRN58	*LcCCDlike-a*	5	21	1331	1352	Translation	2
Lc-miRN58	*LcCCDlike-a*	5	21	3005	3026	Translation	2
Lc-miRN53	*LcCCD4*	4.5	20	1148	1168	Cleavage	1
Lc-miR166a	*LcCCD4*	5	20	1180	1200	Translation	1
Lc-miRN19	*LcCCD4a1*	4.5	20	821	841	Cleavage	1
Lc-miRN23	*LcCCD4a1*	4.5	21	704	725	Cleavage	1
Lc-miRN56a/b	*LcCCD4a1*	4.5	20	1120	1140	Cleavage	1
Lc-miRN19	*LcCCD4a2*	4.5	20	821	841	Cleavage	1
Lc-miRN23	*LcCCD4a2*	4.5	21	704	725	Cleavage	1
Lc-miRN56a/b	*LcCCD4a2*	4.5	20	1120	1140	Cleavage	1
Lc-miRN56a/b	*LcCCD4b*	4.5	20	1099	1119	Cleavage	1
Lc-miRN23	*LcCCD4b*	5	21	683	704	Cleavage	1
Lc-miRN26a/b	*LcCCD4c1*	4	20	882	902	Cleavage	1
Lc-miRN26a/b	*LcCCD4c2*	3.5	20	1707	1727	Cleavage	1
Lc-miR6833	*LcCCD4c2*	4.5	20	450	470	Cleavage	1
Lc-miR171b/c/d/g/h/j/o/sq	*LcCCD4c2*	5	20	472	492	Cleavage	1
Lc-miRN23	*LcCCD4c2*	5	21	692	713	Cleavage	1
Lc-miRN45	*LcCCD4c2*	5	20	1453	1473	Translation	1
Lc-miR156c/r	*LcCCD7*	5	20	1518	1538	Cleavage	1
Lc-miRN34	*LcCCD8a*	5	20	1518	1538	Cleavage	1
Lc-miRN56a/b	*LcCCD8a*	5	20	1621	1642	Cleavage	1
Lc-miR166b/e/f/g/h/i/j/l/m/n/o	*LcCCD8b*	4.5	20	824	844	Cleavage	1
Lc-miR166c/k	*LcCCD8b*	4.5	20	824	844	Cleavage	1
Lc-miRN13	*LcCCD8b*	5	20	873	893	Cleavage	1
Lc-miR156e	*LcNCED1*	4	19	667	686	Cleavage	1
Lc-miR156a/b/o/p/q	*LcNCED1*	5	19	667	686	Cleavage	1
Lc-miR156f	*LcNCED1*	5	20	667	687	Cleavage	1
Lc-miR156k/s	*LcNCED1*	5	19	667	686	Cleavage	1
Lc-miRN16a/b	*LcNCED1*	5	21	412	433	Translation	1
Lc-miRN54a/b	*LcNCED1*	5	20	1016	1036	Translation	1
Lc-miR395a/b/c	*LcNCED2*	4.5	20	948	968	Cleavage	1
Lc-miRN24	*LcNCED2*	5	20	687	707	Cleavage	1
Lc-miRN45	*LcNCED2*	5	20	1241	1261	Cleavage	1
Lc-miRN53	*LcNCED2*	5	20	1654	1674	Translation	1

### *Cis*-regulatory elements analysis of *LcCCO* genes

**Fig. 3:**
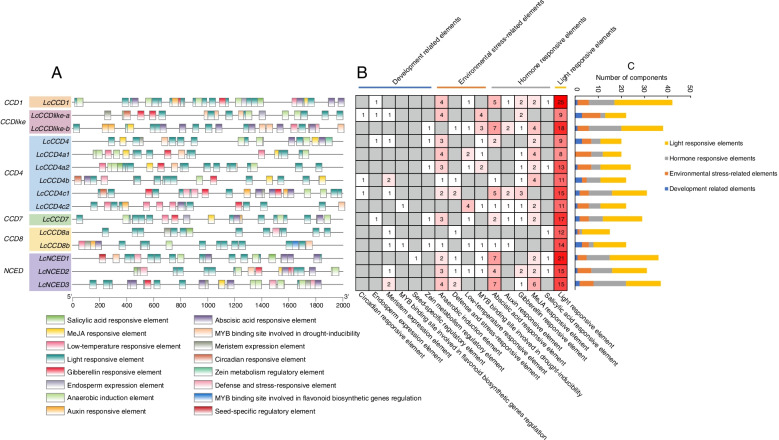
*Cis*-regulatory element analysis of *LcCCO* genes in litchi. **A** The distribution of *cis* regulatory elements on the *LcCCO* gene promoter. **B** and **C** The statistics of *cis* regulatory elements of each *LcCCO* genes.

*Cis*-regulatory elements analysis found that a total of 411 *cis*-elements could be identified in the promoter region of *LcCCO* genes with the exception of common elements like TATA-box and CAAT-box and some unknown functional elements (Fig. 3 and Table S2). Among these elements of *LcCCO* genes, the largest group was light responsive related, included 213 (51.82%) elements, such as Box 4, GA-motif, MRE and G-box elements. The second largest group was about plant hormones related, comprised 103 (25.06%) elements, such as methyl jasmonate (MeJA) response elements (CGTCA-motif and TGACG-motif), salicylic acid (SA) response elements (TCA-element), gibberellin (GA) response elements (GARE-motif, TATC-box and P-box), abscisic acid (ABA) response elements (ABRE) and Auxin responsive element (TGA-element and AuxRR-core). ABA response elements were the largest group of plant hormones related *cis*-elements in the *CCD1*, *CCD4, CCD7, CCD-like,* and *NCED* subfamily. The third largest group was about stress related, embodied 70 (217.03%) elements, such as anaerobic induction element (ARE and GC-motif), defence and stress responsive elements (TC rich repeats) and low temperature responsive elements (LTR). The fourth largest group was about growth and development related, possessed 25 (6.08%) elements, such as endosperm expression (GCN4_motif), meristem expression (CAT-box), MYB binding site involved in flavonoid biosynthesis (MBSI) and seed specific regulatory element (RY-element).

### Structural features of LcCCO proteins

**Fig. 4:**
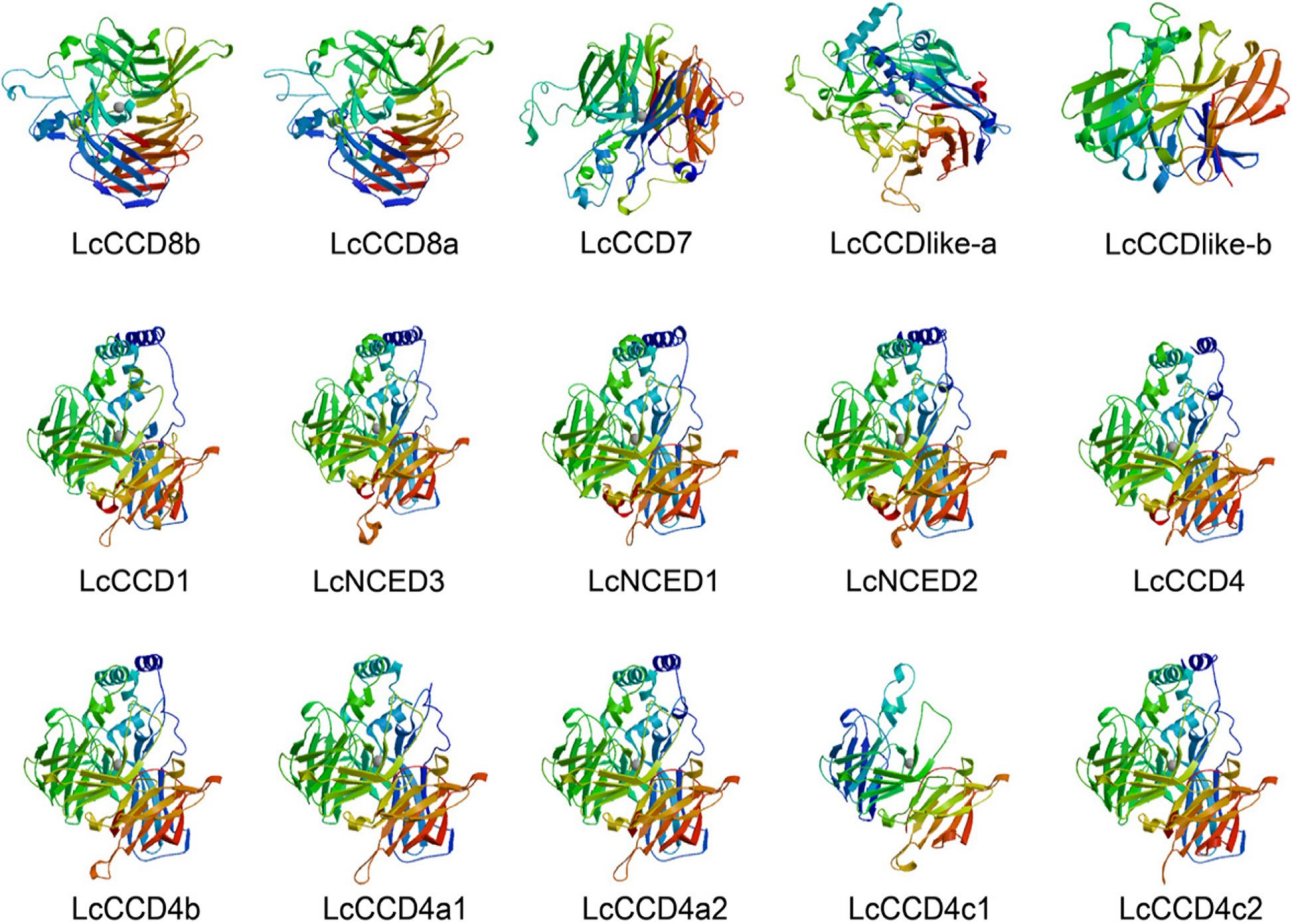
Prediction of three-dimensional domain of LcCCO proteins (purple, blue, green, yellow, orange, red, N-terminal to C-terminal)

Secondary structures analysis showed that LcCCO proteins consisted an α-helix, extended chain and random coil. Random coiled amino acids occupied the largest proportion (> 50%), followed by α-helix (10.31% ~ 28.73%) and extended chain (16.28% ~ 28.08%) (Table S3). 3D structures prediction revealed that the structures of *CCD8* subfamily were similar, the structures of *CCD4* subfamily (excepted for LcCCD4c1 protein), *NCED* subfamily and LcCCD1 protein were similar (Fig. 4), suggested that they shared functionality.

### GO enrichment analysis of *LcCCO* genes

**Fig. 5:**
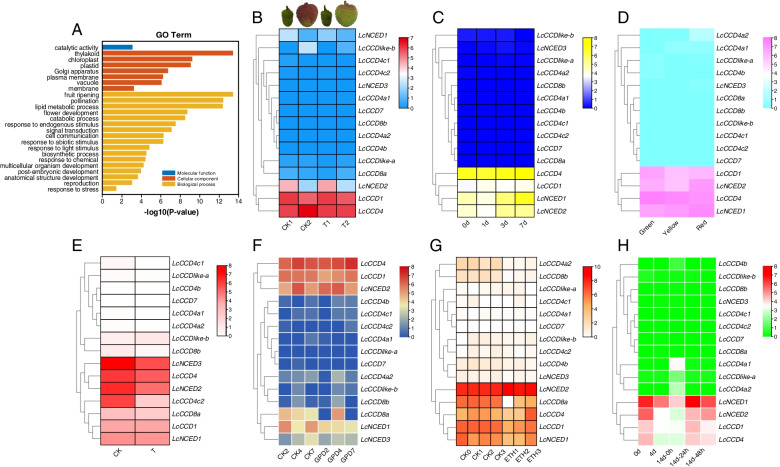
The GO enrichment analysis and expression pattern analysis of *LcCCO* genes by RNA-seq data. **A** The GO enrichment analysis of *LcCCO* genes in litchi. **B** The expression of *LcCCO* genes during peel coloring of ‘Feizixiao’ litchi treated by exogenous CPPU, CK: control group, T: CPPU treatment group. CK1 and T1: Green stage (the peel just completely wraps the pulp, 35d after anthesis), CK2 and T2: The best edible stage of fruit (57d after anthesis). **C** The expression of *LcCCO* genes of ‘Feizixiao’ litchi on the 0, 1, 3 and 7 days after bags removed. 0d: completely green; 1d, only the stipe was colored, 3d: The peel was half colored, 7d: fully colored. **D** The expression of *LcCCO* genes of ‘Nuomici’ Litchi during three different development stages of fruit. Green: the peel is completely green; Yellow: peel yellow; Red: peel red. **E** The expression of *LcCCO* genes of the entire inflorescences samples of ‘Feizixiao’ litchi on 28 days after the uniconazole treatment. CK: control group; T: treatment group. **F** The expression of *LcCCO* genes of fruit samples of ‘Wuye’ litchi after 2, 4 and 7 days treated by girdling plus defoliation. CK: control group, GPD: girdling plus defoliation. **G** The expression of *LcCCO* genes of abscission zone samples of ‘Feizixiao’ litchi after 0, 1, 2, 3 days treated by exogenous ethephon. CK: control group, ETH: exogenous ethephon treatment. **H** The expression of *LcCCO* genes of the peel samples on 0d and 4d after stored at room temperature and 0 h, 24 h and 48 h stored at room temperature after precooling for 14 days

In order to predict the exact functions the litchi genes, GO enrichment analysis of *LcCCO* genes had been conducted in study. The result showed that the function of *LcCCO* genes functioned in moleculler function, cellular component and biological process (Fig. 5A and Table S4). When comes to the biological process, it was clearly that *LcCCO* genes were involving in the process of fruit ripening, pollination, flower development, catabolic process, response to endogenous stimulus, signal transduction, response to abiotic stimulus, response to light stimulus, reproduction so on.

### Expression patterns analysis of *LcCCO* genes by RNA-seq data

In order to investigate the potential function of *LcCCO* genes, the expression pattern of *LcCCO* genes related to peel coloration, fruit abscission, flowering control, and postharvest preservation of litchi were analysed based on the RNA-seq data supplied by our research group (not published) and other groups published online (Fig. 5B-G).

During the peel coloration inhibition experiment of ‘Feizixiao’ litchi induced by exogenous CPPU treatment (Fig. 5B and Table S5), compared to the complete green stage of fruit, *LcCCD1, LcNCED1,* and *LcNCED2* exhibited down-regulated expression in the best edible stage (this stage of ‘Feizixiao’ litchi which existed ‘stay green’ phenomenon) between control and treatment groups, but much more obviously in the treatment groups (decreased by 1.49, 5.44, and 6.24 times in control groups and 1.33, 2.38, and 5.58 times in the treatment groups separately). *LcCCDlike-b* displayed up-regulated expression, increased by 74.88 times in control groups and 50.41 times in the treatment groups separately. *LcCCD4* just showed up-regulated expression in the control groups, but no obvious change in the treatment groups. During the experiment of light-regulated anthocyanin biosynthesis in the peel of ‘Feizixiao’ litchi (Zhang et al., 2016a), *LcCCD1*, *LcCCD4*, *LcNCED1,* and *LcNCED2* showed up-regulated expression and reached the peak on the third day or seventh day after bags removed. No apparent change found in others genes (Fig. 5C and Table S6). These results suggested that *LcCCDlike-b*, *LcCCD1*, *LcCCD4*, *LcNCED1, and LcNCED2* might play an important role in the fruit maturation of ‘Feizixiao’ litchi.

Compared to ‘Feizixiao’ litchi, ‘Nuomici’ litchi fruit could complete coloring [28]. *LcCCD1* and *LcCCD4* exhibited down-regulated expression in the yellow and red stage of fruit, *LcNCED1* showed up-regulated expression after green stage, and reached the peak in the red stage. *LcNCED2* displayed down-regulated expression at yellow stage and up-regulated expression in the red stage (Fig. 5D and Table S7). These finding suggest that *LcCCD1, LcCCD4*, *LcNCED1,* and *LcNCED2* might function during the fruit maturation of ‘Nuomici’ litchi.

Uniconazole treatment of litchi inflorescences can control flowering and improve fruit-setting in litchi [29]. *LcCCD4*, *LcCCD4c2*, *LcCCD8a*, *LcNCED2, and LcNCED3* showed down-regulated expression obviously in the entire inflorescences after uniconazole spraying, decreased by 1.72, 1.53, 15.97, 2.80, and 3.18 times separately. No apparent change found in other genes (Fig. 5E and Table S8), indicated that the above genes might be involved in the flowering control and fruit-setting improvement of litchi.

In the fruitlet samples during fruit abscission of ‘Wuye’ litchi induced by girlding plus defoliation treatment [30], *LcCCD1*, *LcCCD4a2*, *LcCCD8a,* and *LcNCED1* showed down-regulated expression on the second day after treatment, and *LcCCD4a2* decreased most significantly (11.07 times). *LcCCD4* and *LcNCED2* displayed down-regulated expression on the fourth day after treatment, decreased by 1.15 and 1.13 times separately. *LcNCED3* exhibited down-regulated expression on the seventh day after treatment, *LcCCD4* showed up-regulated expression on the seventh day after treatment (Fig. 5F and Table S9). In the abscission zone samples during fruitlet abscission of ‘Feizixiao’ litchi caused by exogenous ethephon treatment [31], *LcCCD4a2*, *LcCCD4b*, L*cCCD8a,* and *LcCCD8b* showed down-regulated expression evidently on the first, second and third day*. LcCCD1*, *LcCCD4,* and *LcNCED1* displayed down-regulated expression on the first and second day and up-regulated expression on the third day after treatment. *LcNCED2* exhibited up-regulated expression evidently during the whole times (Fig. 5G and Table S10). These results indicated the above genes might be related in the fruitlet abscission of litchi.

In the peel samples of ‘Huaizhi’ litchi on 0d and 4d after stored at room temperature and 0 h, 24 h, and 48 h stored at room temperature after precooling for 14 days [32], *LcCCD1, LcCCD4, LcNCED1, and LcNCED2* showed relative higher expression on 0d in the sample which stored at room temperature without precooling treatment, but expression inhibition could be found obviously in the samples which do the precooling treatment. All of the above four genes showed down-regulated expression on 4d after stored at room temperature without precooling treatment, up-regulated expression on 14 h and 48 h after stored at room temperature treated by precooling. It was interesting that *LcCCD4a1* and *LcCCD4a2* showed significantly up-regulated expression in 0 h stored at room temperature after precooling (Fig. 5H and Table S11). These data suggested that the above genes might be involved in the rapid fruit senescence induced by pre-cold storage.

### Identification of expression patterns of *LcCCO* genes by quantitative qRT-PCR

**Fig. 6:**
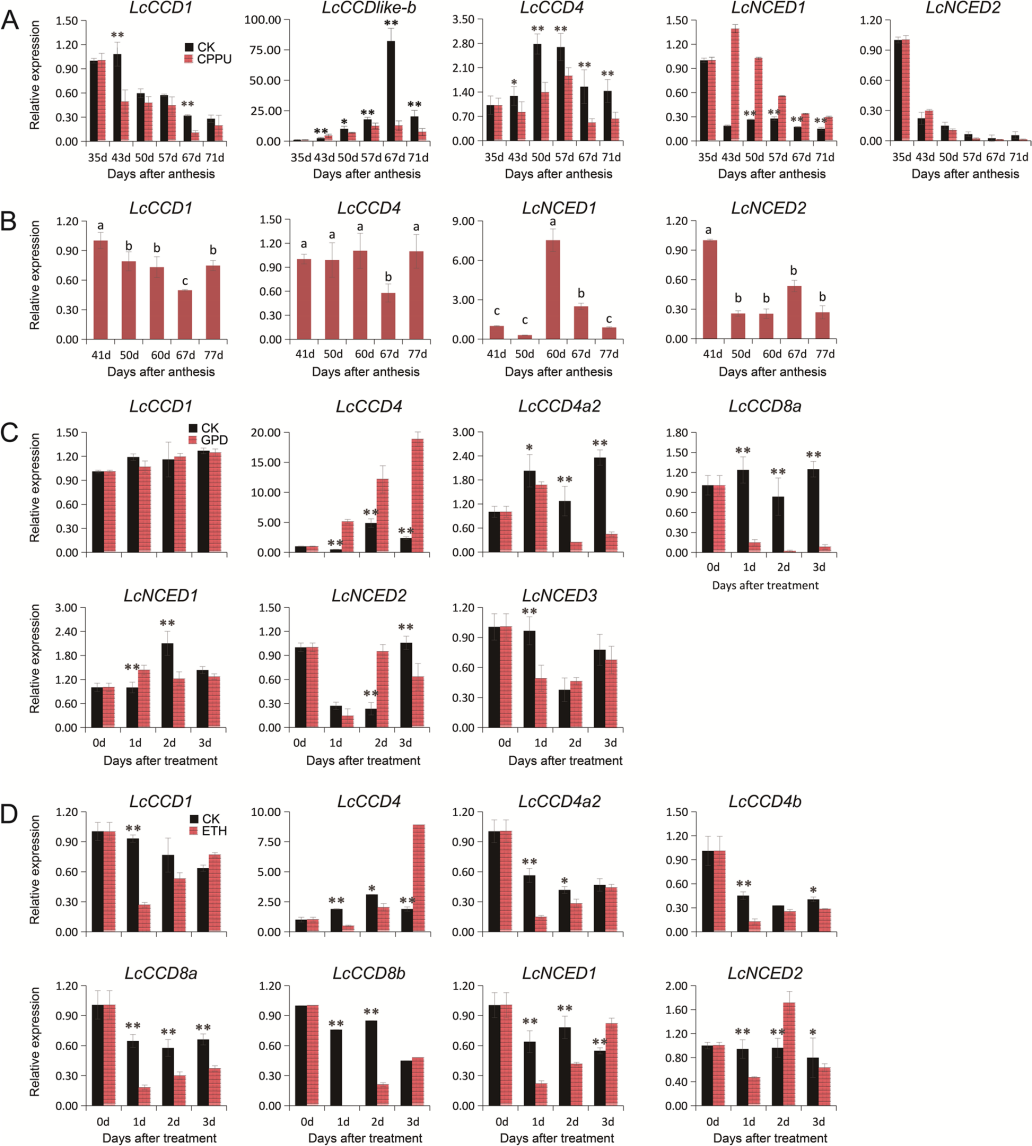
The expression of *LcCCO* genes identified by by qPCR. **A** The expression of *LcCCO* genes of the peel tissues during fruit maturation of ‘Feizixiao’ litchi treated by exogenous CPPU after anthesis. 35d: Green stage (the peel just completely wraps the pulp), corresponding to the CK1 and T1, 57d: The best edible stage of fruit, corresponding to the CK2 and T2 in Fig. 5A. **B** The expression of *LcCCO* genes of the peel tissues during fruit natural maturation of ‘Nuomici’ litchi. **C** The expression of *LcCCO* genes of fruitlet during the fruitlet abscission of ‘Feizixiao’ litchi treated by girdling plus defoliation treatment. **D** The expression of *LcCCO* genes of abscission zone tissues during the fruitlet abscission of ‘Feizixiao’ litchi treated by exogenous ethephon

In order to further explore the potential function of *LcCCO* genes, the samples which involving in the inhibition of peel coloration of ‘Feizixiao’ litchi induced by exogenous CPPU, the natural peel coloration of ‘Nuomici’ litchi, fruitlet abscission of litchi produced by girdling plus defoliation treatment and exogenous ethephon treatment were collected. The expression patterns of these pivotal *LcCCO* genes obtained by the RNA-seq data were assessed by quantitative qRT-PCR (Fig. 6). The results showed that the expression pattern of most of *LcCCO* genes were consistent with their expression patterns in the RNA-seq data described above (Figs. 5B, D, F-G, and 6) excepted for the expression of *LcNCED1* during the coloration of ‘Feizixiao’ litchi and the expression of *LcCCD4* during the coloration of ‘Nuomici’ litchi (Figs. 5B, D, 6A-B). Interestingly, there were also some differences of the expression of *LcCCO* genes between the experiment of fruitlet abscission of ‘Feizixiao’ litchi and ‘Wuye’ litchi produced by girdling plus defoliation treatment (Fig. 6C). This might be caused by the variety differences between ‘Wuye’ litchi and ‘Feizixiao’ litchi. But there were still some *LcCCO* genes shared the similar expression patterns, Such as *LcCCD4*, *LcCCD4a2*, *LcCCD8a, and LcNCED1.*

## Discussion

### Identification of LcCCO genes

Compared to the *MYB*, *bZIP* and *bHLH* gene family, *CCO* is a relatively small gene family in plant. In our study, a total of 15 *LcCCO* genes were identified in litchi and could be divided into six (*CCD1*, *CCD4*, *CCD7*, *CCD8, CCD-like,* and *NCED*) subfamilies based on the phylogenetic relationships analysis with *Arabidopsis thaliana*, *Solanum lycopersicum*, *Malus* × *domestica* and *Litchi chinensis* Sonn (Fig. 1), which was consistent with the previous work [10, 35]. Physicochemical properties analysis showed that the length of most of LcCCO proteins ranged from 500 to 600aa (Table 1), which displayed similarity with other plants [6, 36, 37]. RPE65 domain is a specific conserved domain in CCO protein, which is the key to the enzymatic oxidation activity cleavage of carotenoids [38]. Conserved domain analysis showed that all of LcCCO proteins contained a RPE65 domain and which exhibited similar distribution pattern in the same subfamily. Like distribution of RPE65 domain, gene structure and motif showed high similarity of distribution pattern in the same subfamily too (Fig. 2A-C). These results indicated that the genes in the same subfamily which held the similar function probably. Motif 5 and motif 7 were located in all LcCCO protein, implied that they were important characteristics and may be responsible for common functions between them. Subcellular localization analysis can help to understand the site where the protein will function. In the study, 11 (73.3%) LcCCO proteins were predicted to be located in the chloroplast, suggested that these genes might participate in chlorophyll photosynthesis. 3 (20.0%) LcCCO proteins located in cytoplasm and 1 (6.7%) LcCCO protein located in mitochondria (Table 1), indicated that these genes functioned in cytoplasm and mitochondria, and might not be involved in chlorophyll photosynthesis. These results are consistent with the previous study [35].

MiRNA is considered as a kind of post transcriptional regulator, and play a critical role during the development of plant [39, 39, 40]. In our result, 13 (86.7%) *LcCCO* genes obtained 31 miRNA target sites predicted combined with the litchi miRNAs described previously [41], suggested that post transcriptional regulation of *LcCCO* genes by miRNA might be functioning during the development of litchi. *Cis*-regulatory elements located in the gene promoter region which could regulated the gene expression on transcriptional level [42]. *Cis*-regulatory elements analysis found that a large number of *cis*-elements which involving in light responsive, plant hormones and stress (biological and abiotic stress related) and pant growth and development could be detected (Fig. 3A-C and Table S2). It was interesting that ABRE element which related to ABA response were the largest group in the plant hormones related *cis*-elements. These indicated that the transcription of *LcCCO* genes could be in response to light, plant hormones, biological and abiotic stress and pant growth and development. In order to investigate their function during the development period of litchi, RNA-seq data and quantitative qRT-PCR analyses related to peel coloration, flowering control, fruit abscission, and postharvest preservation were used to do further analysis.

### *LcCCO* genes might be involved in the coloration and flavor of litchi

The colour of horticultural produce, is a key factor that can decide and enhance their economic value. The carotenoids metabolism pathway of CCD4 had been proved to be related to the color formation in plant species like chrysanthemum petalsonly and citrus by affecting the catalytic degradation pathway of carotenoids [14, 16]. ABA was considered as the critical hormone related to the coloration in plant, including strawberry [21], grape [22], sweet cherry [23] and litchi fruit [24]. NCED is a key regulator of ABA biosynthesis. *FaNCED1* RNAi resulted in uncolored strawberry fruits by declining the ABA and anthocyanin content significantly (*Fragaria* × *ananassa*) [20]. ‘Feizixiao’litchi existed in ‘stayed green’category at the best edible stage. RNA-seq data and qRT-PCR analysis showed that the expression of *LcCCD4, LcNCED1,* and *LcNCED2* could be inhibited obviously by exogenous CPPU treatment during fruit maturation of ‘Feizixiao’ litchi. *LcCCD4* reached a peak at 50d and 57d after anthesis (the best edible stage) and then declined, *LcNCED1* and *LcNCED2* reached the peak at 43d and 35d after anthesis separately, but kept relative low expression (Figs. 5B, 6A, Table S5). RNA-seq data showed that *LcCCD4*, *LcNCED1,* and *LcNCED2* exhibited up-regulated expression apparently during light-regulated anthocyanin biosynthesis to promote the coloration of ‘Feizixiao’ litchi after removing bag, and the TPM value of them was much higher than the experiment samples treated by CPPU (Fig. 5B-C and Table S5-6). Compared to ‘Feizixiao’ variety, ‘Nuomici’ litchi fruit could fulfil coloration. RNA-seq data and qRT-PCR analysis showed that *LcCCD4* exhibited relative high expression before 67d after anthesis and reached the peak at 60d (yellow stage). *LcNCED1* exhibited relative higher expression than ‘Feizixiao’ litchi samples described above and reached a peak at 60d after anthesis and then declined, but still kept relative higher expression at 67d (Red stage). *LcNCED2* exhibited two peaks at 41d (green stage) and 67d (red stage) after anthesis (Figs. 5D, 6B and Table S7). Among the above results, the higher expression of *LcCCD4, LcNCED1,* and *LcNCED2* during the early stage of fruit maturation of ‘Nuomici’ litchi might contribute to the carotenoid degradation and anthocyanin accumulation to complete coloration. The relative low expression of *LcCCD4, LcNCED1,* and *LcNCED2* in ‘Feizixiao’ litchi might be the key factor to induce the ‘stay green’ phenomenon. In addition to participating in coloring, *CCO* genes liked *CCD1* was reported to be involving in the formation of the flavor and scent in plants by catalyzing degradation of carotenoids [12, 43]. We could clearly find that *LcCCD1* showed down expression during the fruit maturation in the both of ‘Feizixiao’ and ‘Nuomici’ varieties simultaneously, and *LcCCDlike-b* displayed down expression significantly in ‘Feizixiao’ variety but could not be detected in the ‘Nuomici’ variety (Fig. 5B, D and Table S5, S7). Although the dynamic of carotenoid content all showed a downtrend, but displayed some differences during fruit maturation of ‘Feizixiao’and ‘Nuomici’ varieties (Supplement Fig. 1). These implied that *LcCCDlike-b* might be an important factor which involving in the different flavor between these two varieties. But these conjectures need further works to confirmed them.

### *LcCCO* genes might be involved in fruitlet abscission of litchi

ABA was also reported to be related to plant organ abscission [44, 45]. Litchi is an important subtropical and tropical economic fruit. It is reported that there are 3–5 fruit drop waves (I, II, III, IV, V) between different cultivars, and high ABA lever is regarded one of most important physiological reasons for the fruit drop wave II, III, and V [46]. Based on the public RNA-seq data and qRT-RCR analysis, we found that the expression of *LcNCED1*, *LcNCED2, and* other *LcCCO* genes were enhanced in fruitlet and abscission zone samples during fruitlet abscission induced by GPD and exogenous ETH treatment in ‘Feizixiao’ variety separately (Figs. 5F-G, 6C-D and Table S9-10). These implied that *LcNCED1* and *LcNCED2* might function in accelerating the ABA content to promote the fruitlet abscission of litchi, the function of other *LcCCO genes* needed further work to investigate. Contrary to the result of ‘Feizixiao’ variety, the expression of *LcNCED1*, *LcNCED2* other *CCO* genes in ‘Wuye’ variety were inhibited in the fruitlet samples during fruitlet abscission induced by GPD treatment (Fig. 5F and Table S9). The differences of gene expression between ‘Wuye’ variety and ‘Feizixiao’ variety treated by GPD, which might be caused by the difference between these two varieties, but needed further works to demonstrate this conjecture.

### *LcCCO* genes might be involved in flower control of litchi

Easily flowering and without controlling of the inflorescence development and flowering can lead to low fruit-setting percentage or even zero yield by consuming excessive amounts of accumulated nutrients during the production of litchi [47]. Uniconazole spraying which can regulate the changes of endogenous hormone levels (reducing GA content and increased ABA content) to function in the flower control and fruit retention of litchi, is considered as an effective chemical method [48, 49]. Another research proved that uniconazole was a strong competitive inhibitor of ABA 8'-hydroxylase to effectively inhibit ABA catabolism in Arabidopsis plants [50]. In this study, the RNA-seq data published by Wei et al. [29] showed that *LcNCED2 and LcNCED3* displayed low expression in litchi inflorescences treated by uniconazole (Fig. 5E and Table S8). These suggested that the ABA accumulation is the result of the joint action of biosynthesis regulated by *LcNCED2 and LcNCED3* and catabolism inhibition by uniconazole. The later might be much more important during the flowering controlling to improve the litchi fruit-setting. but also need more experiment to confirm it.

### *LcCCO* genes might be involved in the postharvest preservation of litchi

Litchi is a non-climacteric tropical and subtropical fruit which is highly perishable, and typical symptoms is pericarp browning and loss of flavor after harvest. The shelf life of litchi fruit could be prolonged up to a month storage in cold environment, but the fruit senescence of fruits which were at ambient temperatures after pre-cold storage treatment could be accelerated, only 1–2 days, much less than the fruit under ambient conditions, which is approximately 4–6 days [32, 51, 52]. ABA was considered as one of key factors which contributed the fruit senescence [53, 54]. In this study, The RNA-seq data published by Yun et al. [32] showed that *LcCCD4a1* and *LcCCD4a2* displayed significantly up-regulated expression at 0 h, *LcCCD1, LcCCD4, LcNCED1, and LcNCED2* displayed up-regulation at 14 h and 48 h stored at room temperature after treated by precooling (Fig. 5H and Table S11). These data suggested that the above genes might be involved in the rapid fruit senescence induced by pre-cold storage by enhanced the ABA content and accelerate the carotenoid degradation, but need further experiments to confirmed it.

## Conclusions

In conclusion, *CCO* gene family were identified comprehensively in litchi. A total of 15 *LcCCO* genes were identified and could be classified into five subfamilies based on the phylogenetic relationships with other several species. And then the physicochemical properties, the distribution of gene structure, conserved domain and motif, *cis*-elements, miRNA target sites, 3D protein structure were further analysed. In addition, RNA-seq data and qRT-PCR analysis revealed that *LcCCO* genes might be related to the peel coloration, fruit flavor, flower control, fruit abscission and postharvest storage of litchi. Our result not only will help lay the foundation for the function identification of *CCO* gene family in litchi, but also will help us understand the role of this gene family in other plant species.

## Methods

### Identification of *LcCCO* genes

The genome and gene annotation files of litchi was supplied by College of Horticulture, South China Agricultural University, Guangzhou, China (data unpublished yet). The CCO protein sequences of *Arabidopsis thaliana* were downloaded from TAIR database (https://www.arabidopsis.org/). Homology search was conducted by the TBtools software [26], and then confirmed the RPE65 (retinal pigment epithelial membrane protein) domain used the Pfam database (http://pfam.xfam.org/) and Hmmer2.3 database (http://hmmer.janelia.org/). The sequences did not contain the RPE65 domain would be deleted, and the rest proteins were considered as the litchi CCO (LcCCO) proteins. Physicochemical properties of LcCCO proteins like molecular weight (MW), Pi, instability index, aliphatic index was analyzed by ExPASY website (http://web.expasy.org/protparam/), Subcellular localization prediction was conducted by the BUSCA website (http://busca.biocomp.unibo.it/), signal peptide and transmembrane structure were predicted by the MBC website (http://cello.life.nctu.edu.tw).

### Phylogenetic analysis

The CCO protein sequences of *Arabidopsis thaliana*, *Solanum lycopersicum*, *Malus* × *domestica* and *Litchi chinensis* Sonn. were used to do phylogenetic analysis. Phylogenetic tree was conducted by Clustal X2 and MEGA 6 software using maximum-likelihood (ML) methods with the following parameter settings: poisson model, partial deletion and 1000 bootstrap replicates. At last, the LcCCO proteins were renamed depended on which subfamily they belonged to.

### Gene structure, conserved domain, conserved motif and chromosomal arrangement analysis

The structure information of *LcCCO* genes can be acquired by the gff file of litchi genome. The conserved domain identification was conducted by NCBI cd-search website (https://www.ncbi.nlm.nih.gov/Structure/bwrpsb/bwrpsb.cgi) and Pfam website (http: //www.sanger.ac.uk/software/Pfam/). The motif analysis was executed by MEME tools (http://meme-suite.org/tools/meme). Phylogenetic tree, gene structure, conserved domain and motif of *LcCCO* genes were displayed by TBtools software [26].

### miRNA target site prediction

Litchi miRNAs sequences could be obtained from the previous works (Ma et al., 2018) and the *LcCCO* genes sequences were adopted to do the miRNA target site prediction by the psRNATarget website (https://www.zhaolab.org/psRNATarget/analysis?function=3) with default parameter settings.

### *Cis*-regulatory elements analysis

The 2000 bp upstream sequences from the translation initiation codon ‘ATG’ of *LcCCO* genes were extracted by the TBtools software [26] and then used to do the *cis*-regulatory elements analysis by Plant Care database (http://bioinformatics.psb.ugent.be/webtools/plantcare/html/).

### 3D protein structure analysis

Firstly, protein secondary structure of LcCCO proteins were predicted by GOR IV (https://npsa-prabi.ibcp.fr/cgi-bin/npsa_automat.pl?page=/NPSA/npsa_gor4.html), and then 3D protein structure was predicted by the SWISS-MODEL online tools (https://swissmodel.expasy.org/).

### Gene Ontology (GO) enrichment analysis

Firstly, all litchi genes was blasted to the uniprot_sprot.fasta file downlorded from Swissprot database (https://www.uniprot.org/). Then GO annotation and enrichment analysis was conducted by the TBtools software [26].

### Expression analysis of *LcCCO* genes by RNA-seq data

RNA-seq data in this study used for the expression analysis including the following seven sets of data (The first set data was home data, RNA-seq was conduct by the paied-end sequencing based on Illumina platform. Other six sets data were publically available): (1) The peel samples of complete green stage (the peel just completely wraps the pulp) and the best edible stage of ‘Feizixiao’ variety with three biological repetitions, which obtained from the inhibition of the fruit coloration experiment by exogenous N-(2-Chloro-4-pyridyl)-N’-phenylurea (CPPU) (not published yet). (2) The peel samples of coloration of ‘Feizixiao’ litchi on 0d, 1d, 3d and 7d after removed bag, accession number is SRA312830 (https://www.ncbi.nlm.nih.gov/sra/?term=SRX1445119) [27]. (3) Three stages of peel samples of ‘Nuomici’ litchi: green, yellow and red peel stage, accession number is SRP047115 (https://www.ncbi.nlm.nih.gov/sra/?term=SRP047115) [28]. (4) The entire inflorescences samples on 28 days after the uniconazole treatment, accession number is SRP092890 (https://www.ncbi.nlm.nih.gov/sra/?term=SRP092890) [29]. (5) The fruit samples on 2d, 4d and 7d after fruit abscission of ‘Wuye’ litchi caused by girdling plus defoliation treatment (GPD), accession number is SRA234477 (https://www.ncbi.nlm.nih.gov/sra/?term=SRA234477) [30]. (6) The abscission zone samples on 0d, 1d, 2d and 3d after fruit abscission of ‘Feizixiao’ litchi caused by exogenous ethephon (ETH), accession number is SRP173341 (https://www.ncbi.nlm.nih.gov/sra/?term=SRP173341) [31]. (7) The peel samples on 0d and 4d after stored at room temperature and 0 h, 24 h and 48 h stored at room temperature after precooling for 14 days, accession number is SRA247016 (https://www.ncbi.nlm.nih.gov/sra/?term=SRA247016) [32]. More detailed plant material information were listed in Table 3. The transcripts per kilobase million (TPM) value of *LcCCO* genes were calculated by the Salmon software [33]. The log2 value of TPM were used to draw heatmaps by TBtools software [26], and if the TPM values of were less than 1 in any group samples (such as in control group or treatment group), they will be discarded without further analysis. The *P* value was conducted by the edgeR tools of Cloud platform OmicShare of Genedenovo Biotechnology Co., Ltd (Guangzhou, China) (https://www.omicshare.com/tools/).

**Table 3:** Plant materials used for RNA-Seq and qRT-PCR analysis

Set of RNA-Seq data	**Variety**	**Sample ID and**	**Samples**	**Treatment**	**Platform**	**LibraryLayout**	**Accesion number**	**Data sources**
Plant materials for RNA-Seq analysis								
1	‘Feizixiao’ (27-year-old)	CK1_1, CK1_2,CK1_3, CK2_1, CK2_2,CK2_3, T1_1, T1_2, T1_3, T2_1, T2_1, and T2_1.	CK1 and T1: 35d Green stage (the peel just completely wraps the pulp, 35d after anthesis); CK2 and T2: 57d (The best edible stage of fruit) after anthesis.	35 days after anthesis treated with 4 mg/L CPPU.	ILLUMINA	PAIRED		Home data
2	‘Feizixiao’ (8-year-old)	0d (SRR2952606),1d (SRR2954687), 3d (SRR2954690) and 7d (SRR2954691).	0d (completely green), 1d (only the stipe was colored), 3d (The peel was half colored), and 7d (fully colored).(mix sample of 3 biological replicates).	Fruit clusters were bagged at 42 days after anthesis and removed after 2 weeks.	ILLUMINA	PAIRED	SRA312830	Zhang et al., 2016
3	‘Nuomici’ (Adult tree)	Green (SRX700596),Yellow (SRX700598), and Red (SRX700599).	Green,Yellow and Red represented 52, 62 and 72 days after anthesis separately. (mix sample of 3 biological replicates)	Normal condition.	ILLUMINA	SINGLE	SRP047115	Lai et al., 2015
4	‘Feizixiao’ (10-year-old)	CK (SRX2336010) and T (SRX2934817).	CK and T reprensted 0 and 28 days after the uniconazole treatment. (mix sample of 2 biological replicates).	Inflorescence length of about 15 cm treated with 50 ppm Uniconazole.	ILLUMINA	SINGLE	SRP092890	Wei et al., 2015
5	‘Wuye’ ( 9-year-old)	CK2 (SRX847812), CK4 (SRX847822), CK7 (SRX847823), GPD2 (SRX847824), GPD4 (SRX847825), and GPD7 (SRX847826).	GPD2, GPD4, and GPD7 represented 2, 4 and 7 days after treatment. (Mix sample).	Treated with girdling followed by defoliation (GPD treatment) at 35 days after anthesis	ILLUMINA	SINGLE	SRA234477	Li et al., 2015
6	‘Feizixiao’ (9-year-old)	CK0 (SRX5126892),CK1 (SRX5126893), CK2 (SRX5126894), CK3 (SRX5126895), ETH1 (SRX5126896), ETH2 (SRX5126897), and ETH3(SRX5126898).	CK0,CK1,CK2, and CK3 represented 0, 1,2 and 3 days after treatment	Treatment 250 mg/L ethephon solution at 25 days after anthesis.	ILLUMINA	SINGLE	SRP173341	Li et al., 2015
7	‘Feizixiao’ (Adult tree)	0d (SRX968371), 4d (SRX968373), 14d-0 h (SRX968375), 14d-24 h (SRX968377), and 14d-48 h (SRX968379).	Samples after harvest on 0d and 4d after stored at room temperature and 0 h, 24 h, and 48 h stored at room temperature after precooling for 14 days. (1 biological replicate).	Samples after harvest.	ILLUMINA	SINGLE	SRA247016	Yun et al., 2015
Plant materials for qRT-PCR analysis								
1	‘Feizixiao’ (27-year-old)	35d, 43d, 50d, 57d, 67d, and 71d.	Peel (3 biological replicates).	35 days after anthesis treated with 4 mg/L CPPU.				Home data
2	‘Nuomici’ (27-year-old)	41d, 50d, 60d, 67d, and 77d.	Peel (3 biological replicates).	Normal condition, ( sample follected at 41 days after anthesis).				Home data
3	‘Feizixiao’ (27-year-old)	0d, 1d, 2d, and 3d.	Fruitlet (3 biological replicates).	Girdling plus defoliation treatment (GPD) at 35 days after anthesis.				Home data
4	‘Feizixiao’ (27-year-old)	0d, 1d, 2d, and 3d.	Abscission zone tissues (3 biological replicates).	250 mg/L Exogenous ethephon (ETH) at 35 days after anthesis.				Home data

### Expression analysis of *LcCCO* genes identified by quantitative qRT-PCR

To further investigate the function of *LcCCO* genes, four sets of litchi samples with 3 biological replicates in each group were collected for the quantitative qRT-PCR analysis as followed: (1) The peel samples of different development stages (on 35d (complete green stage, corresponed to the T1 and CK1 treatment used in the RNA-seq data), 43d, 50d, 57d (best edible stage, corresponed to the T2 and CK2 treatment used in the RNA-seq data), 67d and 71d after anthesis) of ‘Feizixiao’ litchi fruit after exogenous CPPU treatment. (2) The peel samples of different development stages of ‘Nuomici’litchi (on 41d (green stage), 50d (early yellow stage), 60d (late yellow stage), 67d (best edible stage, red stage) and 77d after anthesis, corresponed to the RNA-seq data above, including green, yellow and red stage). (3) The fruitlet samples of different stage on 0d, 1d, 2, d and 3d of ‘Feizixiao’ litchi after GPD treatment. (4) The Absicssion zone samples of different stage on 0d, 1d, 2, d and 3d of ‘Feizixiao’ litchi after exogenous ETH treatment. More detailed plant material information were listed in Table 3. Total RNA extracted by the RNA Kit RNAiso Plus (#9108) and Fruit-mate (#9192) from Takara Biomedical Technology (Beijing) Co., Ltd, China. The quantity and quality of RNA were conducted by the NanoPhotometer spectrophotometer (#Nano-600) from Jinpeng Analytical Instrument Co., Ltd (Shanghai), China). Reverse transcription and qRT-PCR conducted by the following kits: HiScript III 1st Srand cDNA Synthesis Kit (R312) and ChamQ Universal SYBR qRT-PCR Master Mix (Q711) from Vazyme Biotech Co.,Ltd, Nanjing, China separately. *EF-1α* and *GAPDH* genes were used as reference genes [34]. All the primers were listed in Table S1. The 2^−ΔΔCt^ method was adopted to do result calculation. All the samples were set three technical repetitions. T-test was adopted to do difference analysis.

## Supplementary Information

**Additional file 1. Supplement Figure 1.** The dynamic of carotenoid content during fruit maturation of litchi. A: ‘Feizixiao’ variety; B: ‘Nuomici’ variety. Different letters indicated statistically differences between days of after anthesis using one-way ANOVA with the SAS test (*P *<0.05). **Supplement Table S1.** Primers of selected *LcCCO *genes in litchi and reference genes. **Supplement Table S2.** *Cis*-acting element information in the promoter region of *LcCCO *genes in Litchi. **Supplement Table S3.** Two-dimensional structures of LcCCO proteins. **Supplement Table S4.** GO enrichment analysis of *LcCCO *genes. **Supplement Table S5.** The TPM value and differential expression analysis of *LcCCO *genes during pericarp coloring of ‘Feizixiao’ litchi treated by exogenous CPPU. **Supplement Table S6.** The TPM value and differential expression analysis of *LcCCO *genes of ‘Feizixiao’ litchi on the 0, 1, 3 and 7 days after bags removed (Zhang et al., 2016a). **Supplement Table S7.** The TPM value and differential expression analysis of *LcCCO *genes in ‘Nuomici’ Litchi during three different development stages of fruit (Lai et al., 2015). **Supplement Table S8.** The TPM value and differential expression analysis of *LcCCO *genes of the entire inflorescences samples of 'Feizixiao’ litchi on 28 days after the uniconazole treatment (Wei et al., 2017b). **Supplement Table S9.** The TPM value and differential expression analysis of *LcCCO *genes of fruit samples of ‘Wuye’ litchi after 2, 4 and 7 days treated by girdling plus defoliation(Li et al., 2015a). **Supplement Table S10.** The TPM value and differential expression analysis of *LcCCO *genes of abscission zone samples of ‘Feizixiao’litchi after 0, 1, 2, 3 days treated by exogenous ethephon (Li et al., 2015b). **Supplement Table S11.** The TPM value and differential expression analysis of *LcCCO *genes of the peel samples on 0d and 4d after stored at room temperature and 0h, 24h and 48h stored at room temperature after precooling for 14 days [32].

## Data Availability

The RNA-seq data are obtained from NCBI (https://www.ncbi.nlm.nih.gov/Traces/study/), and the accession numbers are SRA312830, SRP047115, SRP092890, SRA234477, SRP173341 and SRA247016. The COO protein sequences The CCO protein sequences of *Arabidopsis thaliana* were downloaded from TAIR database (https://www.arabidopsis.org/). The litchi genome data used in this study had been deposited into CNGB Sequence Archive (CNSA, https://db.cngb.org/cnsa/) of China National GeneBank DataBase (CNGBdb) with accession number CNP0001024, which will be released after the publication of the litchi genome paper. Public access to all databases is open. Other data supporting the results of this article are included within the article and its additional files.

## Abbreviations

CCO: Carotenoid cleavage oxygenase
CCD: Carotenoid cleavage dioxygenase
NCED: 9-Cis-epoxycarotenoid dioxygenase
CPPU: N-(2-Chloro-4-pyridyl)-N’-phenylurea
ABA: Abscisic acid
GPD: Girdling plus defoliation treatment
ETH: Ethephon
NCBI: National Center for Biotechnology Information
MW: Molecular weight
pI: Isoelectric point
GRAVY: Grand average of hydropathicity
qRT-PCR: Real-time polymerase chain reaction
EF-1α: Elongation factor 1-alpha
GAPDH: Glyceraldehyde-3-phosphate dehydrogenase
