# Supplementary material for: Genome-wide identification and expression analysis of carotenoid cleavage oxygenase genes in Litchi (Litchi chinensis Sonn.)
Source: BMC Plant Biol. 2022 Aug 9;22:394. doi: 10.1186/s12870-022-03772-w (PMC9361530; doi:10.1186/s12870-022-03772-w)
Supplement: Supplementary file 1 — Additional file 1. Supplement Figure 1. The dynamic of carotenoid content during fruit maturation of litchi. A: ‘Feizixiao’ variety; B: ‘Nuomici’ variety. Different letters indicated statistically differences between days of after anthesis using one-way ANOVA with the SAS test (P <0.05). Supplement Table S1. Primers of selected LcCCO genes in litchi and reference genes. Supplement Table S2. Cis-acting element information in the promoter region of LcCCO genes in Litchi. Supplement Table S3. Two-dimensional structures of LcCCO proteins. Supplement Table S4. GO enrichment analysis of LcCCO genes. Supplement Table S5. The TPM value and differential expression analysis of LcCCO genes during pericarp coloring of ‘Feizixiao’ litchi treated by exogenous CPPU. Supplement Table S6. The TPM value and differential expression analysis of LcCCO genes of ‘Feizixiao’ litchi on the 0, 1, 3 and 7 days after bags removed (Zhang et al., 2016a). Supplement Table S7. The TPM value and differential expression analysis of LcCCO genes in ‘Nuomici’ Litchi during three different development stages of fruit (Lai et al., 2015). Supplement Table S8. The TPM value and differential expression analysis of LcCCO genes of the entire inflorescences samples of 'Feizixiao’ litchi on 28 days after the uniconazole treatment (Wei et al., 2017b). Supplement Table S9. The TPM value and differential expression analysis of LcCCO genes of fruit samples of ‘Wuye’ litchi after 2, 4 and 7 days treated by girdling plus defoliation(Li et al., 2015a). Supplement Table S10. The TPM value and differential expression analysis of LcCCO genes of abscission zone samples of ‘Feizixiao’litchi after 0, 1, 2, 3 days treated by exogenous ethephon (Li et al., 2015b). Supplement Table S11. The TPM value and differential expression analysis of LcCCO genes of the peel samples on 0d and 4d after stored at room temperature and 0h, 24h and 48h stored at room temperature after precooling for 14 days [32]. [file 12870_2022_3772_MOESM1_ESM.pdf]

Supplement Figure 1. The dynamic of carotenoid content during fruit maturation of litchi. A: 'Feizixiao' variety; B: 'Nuomici' variety. Different letters indicated statistically differences between days of after anthesis using one-way ANOVA with the SAS test (*P* <0.05).

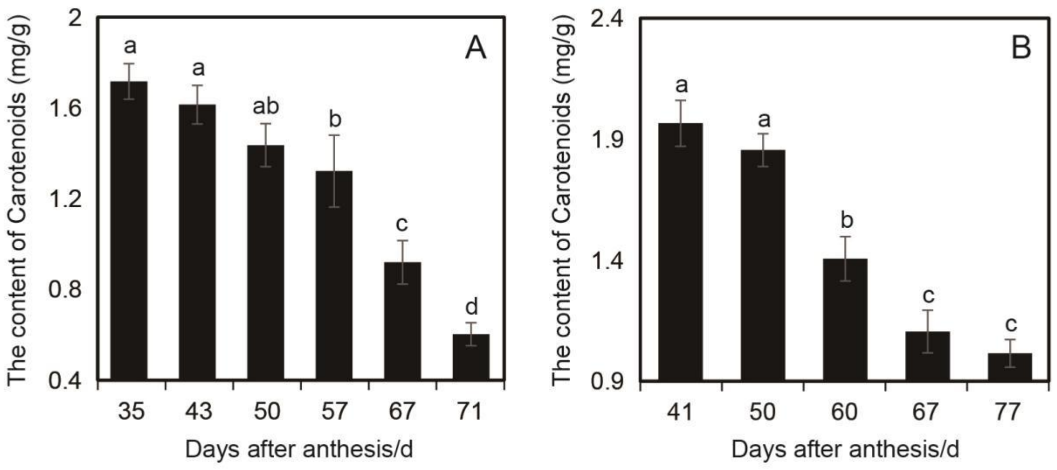

Supplement Table S1. Primers of selected *LcCCO* genes in litchi and reference genes.

Gene name	Primer_F(5' to 3')	Primer_R(5' to 3')
LcEF-1α	CGTGGATTCGTCGCATCTAAC	CCGCCTGTCAATCTTGGTCAT
LcGAPDH	GATACAGTTCCCGTGTTGTTGAC	CATAAAGACACATAACACCACACTC
LcCCD1	CGTCCAGTTTTCTCCACCCA	ATCCTCTCCGTGGTTCTGGA
LcCCDlike-b	TGGGGATGCGTGTAACCAAA	GGCCTCCAAGAACAACCCTG
LcCCD4	TGTGTATGCAGCAGTGGGAG	ACCCATCATCCTCCTCAGCT
LcCCD4a2	ACCACTCCGTCTCTCCAGAT	CCGGTGAAAACATGGCTTGG
LcCCD4b	TCCATCCGTTGATCCAAGCC	TGAAGAGAGTTGCGAGCTGG
LcCCD8a	AGTTTGAGTGGTGCCCGAAA	GGCCGTCAATCTCCCTTCAT
LcCCD8b	GTTCTCCGAGGTCCCCAAAG	CCTATCGTCTCCAGCGTGTC
LcNCED1	TGAACCGAAACATGCTGGGA	AAAACAAAGGCTCCCCACCA
LcNCED2	TGGTCACCACTTCTTCGACG	CAAGAGCCGACCAATCCCAT
LcNCED3	CGGGTAACTTCGCTCCTGTT	GCCATTCAGACAGGTTGGGA

Supplement Table S2. *Cis*-acting element information in the promoter region of *LcCCO* genes in Litchi.

Gene name	*Cis*-acting elements name	Core sequence	Position	Length	Species	Type
LcCCD1	ARE	AAACCA	53	6	Zea mays	Light responsive element
LcCCD1	ATCT-motif	AATCTAATCC	36	10	Pisum sativum	Light responsive element
LcCCD1	Box 4	ATTAAT	633	6	Petroselinum crispum	Light responsive element
LcCCD1	Box 4	ATTAAT	911	6	Petroselinum crispum	Light responsive element
LcCCD1	Box 4	ATTAAT	1307	6	Petroselinum crispum	Light responsive element
LcCCD1	Box 4	ATTAAT	1343	6	Petroselinum crispum	Light responsive element
LcCCD1	CAG-motif	GAAAGGCAGAC	1126	10	Arabidopsis thaliana	Light responsive element
LcCCD1	CGTCA-motif	CGTCA	1727	5	Hordeum vulgare	Light responsive element
LcCCD1	G-Box	CACGTG	1912	6	Pisum sativum	Light responsive element
LcCCD1	G-Box	CACGTT	1984	6	Pisum sativum	Light responsive element
LcCCD1	G-box	TACGTG	460	6	Arabidopsis thaliana	Light responsive element
LcCCD1	G-box	ACACGTGT	1911	8	Brassica napus	Light responsive element
LcCCD1	G-box	CACGTG	1912	6	Arabidopsis thaliana	Light responsive element
LcCCD1	GCN4_motif	TGAGTCA	1259	7	Oryza sativa	Light responsive element
LcCCD1	GT1-motif	GGTTAAT	607	7	Avena sativa	Light responsive element
LcCCD1	GT1-motif	GGTTAA	608	6	Arabidopsis thaliana	Light responsive element
LcCCD1	GT1-motif	GGTTAA	738	6	Arabidopsis thaliana	Light responsive element

Gene name	*Cis*-acting elements name	Core sequence	Position	Length	Species	Type
LcCCD1	GT1-motif	GGTTAA	968	6	Arabidopsis thaliana	Light responsive element
LcCCD1	GT1-motif	GGTTAAT	1687	7	Avena sativa	Light responsive element
LcCCD1	GT1-motif	GGTTAA	1762	6	Arabidopsis thaliana	Light responsive element
LcCCD1	I-box	cCATATCCAAT	801	10	Flaveria trinervia	Light responsive element
LcCCD1	LAMP-element	CTTTATCA	386	8	Pisum sativum	Light responsive element
LcCCD1	LTR	CCGAAA	1644	6	Hordeum vulgare	Light responsive element
LcCCD1	MRE	AACCTAA	1684	7	Petroselinum crispum	Light responsive element
LcCCD1	P-box	CCTTTTG	773	7	Oryza sativa	Light responsive element
LcCCDlike-a	AE-box	AGAAACTT	1710	8	Arabidopsis thaliana	Light responsive element
LcCCDlike-a	ATC-motif	AGTAATCT	1021	8	Spinacia oleracea	Light responsive element
LcCCDlike-a	ATCT-motif	AATCTAATCC	659	9	Pisum sativum	Light responsive element
LcCCDlike-a	GATA-motif	AAGGATAAGG	1285	9	Solanum tuberosum	Light responsive element
LcCCDlike-a	GT1-motif	GGTTAA	1974	6	Arabidopsis thaliana	Light responsive element
LcCCDlike-a	I-box	gGATAAGGTG	1153	9	Zea mays	Light responsive element
LcCCDlike-a	I-box	AGATAAGG	1155	8	Triticum aestivum	Light responsive element
LcCCDlike-a	I-box	AGATAAGG	1285	8	Triticum aestivum	Light responsive element
LcCCDlike-a	TCT-motif	TCTTAC	1865	6	Arabidopsis thaliana	Light responsive element
LcCCDlike-b	G-box	CACGAC	246	6	Zea mays	Light responsive element
LcCCDlike-b	G-box	CACGTC	667	6	Zea mays	Light responsive element

Gene name	*Cis*-acting elements name	Core sequence	Position	Length	Species	Type
LcCCDlike-b	G-box	CCACGTAA	810	8	Brassica napus	Light responsive element
LcCCDlike-b	G-box	TACGTG	811	6	Arabidopsis thaliana	Light responsive element
LcCCDlike-b	G-box	TACGTG	1080	6	Arabidopsis thaliana	Light responsive element
LcCCDlike-b	G-box	TACGTG	1325	6	Arabidopsis thaliana	Light responsive element
LcCCDlike-b	G-box	CACGTC	1770	6	Zea mays	Light responsive element
LcCCDlike-b	G-Box	CACGTT	1533	6	Pisum sativum	Light responsive element
LcCCDlike-b	GT1-motif	GGTTAA	630	6	Arabidopsis thaliana	Light responsive element
LcCCDlike-b	Box 4	ATTAAT	27	6	Petroselinum crispum	Light responsive element
LcCCDlike-b	Box 4	ATTAAT	602	6	Petroselinum crispum	Light responsive element
LcCCDlike-b	Box 4	ATTAAT	617	6	Petroselinum crispum	Light responsive element
LcCCDlike-b	Box 4	ATTAAT	909	6	Petroselinum crispum	Light responsive element
LcCCDlike-b	Box 4	ATTAAT	1286	6	Petroselinum crispum	Light responsive element
LcCCDlike-b	chs-CMA2a	TCACTTGA	1003	8	Petroselinum crispum	Light responsive element
LcCCDlike-b	GATA-motif	AAGGATAAGG	1695	9	Solanum tuberosum	Light responsive element
LcCCDlike-b	I-box	GTATAAGGCC	267	9	Larix laricina	Light responsive element
LcCCDlike-b	TCT-motif	TCTTAC	593	6	Arabidopsis thaliana	Light responsive element
LcCCD4	G-box	TACGTG	1619	6	Arabidopsis thaliana	Light responsive element
LcCCD4	G-box	TACGTG	1844	6	Arabidopsis thaliana	Light responsive element
LcCCD4	G-Box	TCCACATGGCA	466	10	Triticum aestivum	Light responsive element

Gene name	*Cis*-acting elements name	Core sequence	Position	Length	Species	Type
LcCCD4	GT1-motif	GGTTAA	897	6	Arabidopsis thaliana	Light responsive element
LcCCD4	Box 4	ATTAAT	1275	6	Petroselinum crispum	Light responsive element
LcCCD4	GA-motif	ATAGATAA	736	8	Arabidopsis thaliana	Light responsive element
LcCCD4	GA-motif	ATAGATAA	796	8	Arabidopsis thaliana	Light responsive element
LcCCD4	TCT-motif	TCTTAC	285	6	Arabidopsis thaliana	Light responsive element
LcCCD4	MRE	AACCTAA	1678	7	Petroselinum crispum	Light responsive element
LcCCD4a1	GT1-motif	GGTTAA	883	6	Arabidopsis thaliana	Light responsive element
LcCCD4a1	Box 4	ATTAAT	867	6	Petroselinum crispum	Light responsive element
LcCCD4a1	Box 4	ATTAAT	1567	6	Petroselinum crispum	Light responsive element
LcCCD4a1	TCT-motif	TCTTAC	567	6	Arabidopsis thaliana	Light responsive element
LcCCD4a1	AT1-motif	AATTATTTTTTATT	1545	13	Solanum tuberosum	Light responsive element
LcCCD4a1	AE-box	AGAAACTT	1696	8	Arabidopsis thaliana	Light responsive element
LcCCD4a1	MRE	AACCTAA	467	7	Petroselinum crispum	Light responsive element
LcCCD4a1	MRE	AACCTAA	481	7	Petroselinum crispum	Light responsive element
LcCCD4a2	AAAC-motif	CAATCAAAACCT	1669	11	Spinacia oleracea	Light responsive element
LcCCD4a2	GT1-motif	GGTTAA	831	6	Arabidopsis thaliana	Light responsive element
LcCCD4a2	Sp1	GGGCGG	1819	6	Oryza sativa	Light responsive element
LcCCD4a2	MRE	AACCTAA	411	7	Petroselinum crispum	Light responsive element
LcCCD4a2	MRE	AACCTAA	425	7	Petroselinum crispum	Light responsive element

Gene name	*Cis*-acting elements name	Core sequence	Position	Length	Species	Type
LcCCD4a2	ATCT-motif	AATCTAATCC	390	9	Pisum sativum	Light responsive element
LcCCD4a2	Box 4	ATTAAT	815	6	Petroselinum crispum	Light responsive element
LcCCD4a2	Box 4	ATTAAT	990	6	Petroselinum crispum	Light responsive element
LcCCD4a2	Box 4	ATTAAT	994	6	Petroselinum crispum	Light responsive element
LcCCD4a2	Box 4	ATTAAT	1184	6	Petroselinum crispum	Light responsive element
LcCCD4a2	TCT-motif	TCTTAC	1063	6	Arabidopsis thaliana	Light responsive element
LcCCD4a2	AT1-motif	AATTATTTTTTATT	1162	13	Solanum tuberosum	Light responsive element
LcCCD4a2	AE-box	AGAAACTT	1313	8	Arabidopsis thaliana	Light responsive element
LcCCD4b	G-Box	CACGTT	108	6	Pisum sativum	Light responsive element
LcCCD4b	Sp1	GGGCGG	1725	6	Oryza sativa	Light responsive element
LcCCD4b	MRE	AACCTAA	477	7	Petroselinum crispum	Light responsive element
LcCCD4b	Box 4	ATTAAT	687	6	Petroselinum crispum	Light responsive element
LcCCD4b	Box 4	ATTAAT	702	6	Petroselinum crispum	Light responsive element
LcCCD4b	Box 4	ATTAAT	747	6	Petroselinum crispum	Light responsive element
LcCCD4b	Box 4	ATTAAT	1304	6	Petroselinum crispum	Light responsive element
LcCCD4b	Box 4	ATTAAT	1539	6	Petroselinum crispum	Light responsive element
LcCCD4b	chs-CMA1a	TTACTTAA	622	8	Daucus carota	Light responsive element
LcCCD4b	I-box	GTATAAGGCC	149	9	Larix laricina	Light responsive element
LcCCD4b	TCT-motif	TCTTAC	626	6	Arabidopsis thaliana	Light responsive element

Gene name	*Cis*-acting elements name	Core sequence	Position	Length	Species	Type
LcCCD4c1	G-Box	CACGTG	1376	6	Pisum sativum	Light responsive element
LcCCD4c1	G-Box	CACGTT	1475	6	Pisum sativum	Light responsive element
LcCCD4c1	G-Box	CACGTT	1613	6	Pisum sativum	Light responsive element
LcCCD4c1	G-box	CACGTG	1376	6	Arabidopsis thaliana	Light responsive element
LcCCD4c1	3-AF1 binding site	TAAGAGAGGAA	871	10	Solanum tuberosum	Light responsive element
LcCCD4c1	Box 4	ATTAAT	596	6	Petroselinum crispum	Light responsive element
LcCCD4c1	Box 4	ATTAAT	612	6	Petroselinum crispum	Light responsive element
LcCCD4c1	Box 4	ATTAAT	806	6	Petroselinum crispum	Light responsive element
LcCCD4c1	Box 4	ATTAAT	810	6	Petroselinum crispum	Light responsive element
LcCCD4c1	Box 4	ATTAAT	1156	6	Petroselinum crispum	Light responsive element
LcCCD4c1	chs-CMA2a	TCACTTGA	1931	8	Petroselinum crispum	Light responsive element
LcCCD4c1	LAMP-element	CTTTATCA	208	8	Pisum sativum	Light responsive element
LcCCD4c1	TCCC-motif	TCTCCCT	282	7	Spinacia oleracea	Light responsive element
LcCCD4c1	TCCC-motif	TCTCCCT	1964	7	Spinacia oleracea	Light responsive element
LcCCD4c1	TCT-motif	TCTTAC	898	6	Arabidopsis thaliana	Light responsive element
LcCCD4c2	G-box	CACGTC	799	6	Zea mays	Light responsive element
LcCCD4c2	GT1-motif	GGTTAAT	364	7	Avena sativa	Light responsive element
LcCCD4c2	GT1-motif	GGTTAA	365	6	Arabidopsis thaliana	Light responsive element
LcCCD4c2	MRE	AACCTAA	1284	7	Petroselinum crispum	Light responsive element

**Gene name**	***Cis*-acting elements name**	**Core sequence**	**Position**	**Length**	**Species**	**Type**
LcCCD4c2	Box 4	ATTAAT	156	6	Petroselinum crispum	Light responsive element
LcCCD4c2	Box 4	ATTAAT	305	6	Petroselinum crispum	Light responsive element
LcCCD4c2	GA-motif	ATAGATAA	866	8	Arabidopsis thaliana	Light responsive element
LcCCD4c2	I-box	AAGATAAGGCT	1410	10	Gossypium hirsutum	Light responsive element
LcCCD4c2	I-box	AGATAAGG	1411	8	Triticum aestivum	Light responsive element
LcCCD4c2	L-box	ATCCCACCTAC	808	10	Petroselinum crispum	Light responsive element
LcCCD4c2	TCCC-motif	TCTCCCT	1843	7	Spinacia oleracea	Light responsive element
LcCCD7	G-box	CACGAC	1181	6	Zea mays	Light responsive element
LcCCD7	G-box	CCACGTAA	1212	8	Brassica napus	Light responsive element
LcCCD7	G-box	TACGTG	1213	6	Arabidopsis thaliana	Light responsive element
LcCCD7	G-box	TACGTG	1827	6	Arabidopsis thaliana	Light responsive element
LcCCD7	ATCT-motif	AATCTAATCC	467	10	Pisum sativum	Light responsive element
LcCCD7	ATCT-motif	AATCTAATCC	477	10	Pisum sativum	Light responsive element
LcCCD7	ATCT-motif	AATCTAATCC	505	9	Pisum sativum	Light responsive element
LcCCD7	Box 4	ATTAAT	50	6	Petroselinum crispum	Light responsive element
LcCCD7	Box 4	ATTAAT	460	6	Petroselinum crispum	Light responsive element
LcCCD7	Box 4	ATTAAT	464	6	Petroselinum crispum	Light responsive element
LcCCD7	Box 4	ATTAAT	567	6	Petroselinum crispum	Light responsive element
LcCCD7	Box 4	ATTAAT	1394	6	Petroselinum crispum	Light responsive element

Gene name	*Cis*-acting elements name	Core sequence	Position	Length	Species	Type
LcCCD7	Box 4	ATTAAT	1509	6	Petroselinum crispum	Light responsive element
LcCCD7	Box 4	ATTAAT	1562	6	Petroselinum crispum	Light responsive element
LcCCD7	Box 4	ATTAAT	1736	6	Petroselinum crispum	Light responsive element
LcCCD7	GATA-motif	AAGATAAGATT	398	10	Arabidopsis thaliana	Light responsive element
LcCCD7	TCT-motif	TCTTAC	1824	6	Arabidopsis thaliana	Light responsive element
LcCCD8a	GT1-motif	GGTTAAT	1797	7	Avena sativa	Light responsive element
LcCCD8a	GT1-motif	GGTTAA	1798	6	Arabidopsis thaliana	Light responsive element
LcCCD8a	GT1-motif	GGTTAA	1881	6	Arabidopsis thaliana	Light responsive element
LcCCD8a	ATCT-motif	AATCTAATCC	1431	9	Pisum sativum	Light responsive element
LcCCD8a	Box 4	ATTAAT	240	6	Petroselinum crispum	Light responsive element
LcCCD8a	Box 4	ATTAAT	460	6	Petroselinum crispum	Light responsive element
LcCCD8a	Box 4	ATTAAT	501	6	Petroselinum crispum	Light responsive element
LcCCD8a	Box 4	ATTAAT	748	6	Petroselinum crispum	Light responsive element
LcCCD8a	Box 4	ATTAAT	815	6	Petroselinum crispum	Light responsive element
LcCCD8a	chs-CMA1a	TTACTTAA	1228	8	Daucus carota	Light responsive element
LcCCD8a	chs-CMA1a	TTACTTAA	1555	8	Daucus carota	Light responsive element
LcCCD8a	AE-box	AGAAACAA	1518	8	Arabidopsis thaliana	Light responsive element
LcCCD8b	G-Box	CACGTT	188	6	Pisum sativum	Light responsive element
LcCCD8b	Sp1	GGGCGG	543	6	Oryza sativa	Light responsive element

Gene name	*Cis*-acting elements name	Core sequence	Position	Length	Species	Type
LcCCD8b	Box 4	ATTAAT	4	6	Petroselinum crispum	Light responsive element
LcCCD8b	Box 4	ATTAAT	364	6	Petroselinum crispum	Light responsive element
LcCCD8b	Box 4	ATTAAT	521	6	Petroselinum crispum	Light responsive element
LcCCD8b	Box 4	ATTAAT	1085	6	Petroselinum crispum	Light responsive element
LcCCD8b	Box 4	ATTAAT	1118	6	Petroselinum crispum	Light responsive element
LcCCD8b	Box 4	ATTAAT	1198	6	Petroselinum crispum	Light responsive element
LcCCD8b	Box 4	ATTAAT	1697	6	Petroselinum crispum	Light responsive element
LcCCD8b	chs-CMA1a	TTACTTAA	1251	8	Daucus carota	Light responsive element
LcCCD8b	GA-motif	ATAGATAA	1599	8	Arabidopsis thaliana	Light responsive element
LcCCD8b	TCT-motif	TCTTAC	183	6	Arabidopsis thaliana	Light responsive element
LcCCD8b	TCT-motif	TCTTAC	527	6	Arabidopsis thaliana	Light responsive element
LcCCD8b	AE-box	AGAAACAA	957	8	Arabidopsis thaliana	Light responsive element
LcNCED1	G-box	CACGAC	442	6	Zea mays	Light responsive element
LcNCED1	G-box	TAAACGTG	1002	8	Brassica oleracea	Light responsive element
LcNCED1	G-box	GCCACGTGGA	1562	9	Arabidopsis thaliana	Light responsive element
LcNCED1	G-box	CACGTG	1564	6	Arabidopsis thaliana	Light responsive element
LcNCED1	G-box	CACGTC	1609	6	Zea mays	Light responsive element
LcNCED1	G-box	CACGTG	1804	6	Arabidopsis thaliana	Light responsive element
LcNCED1	G-Box	CACGTT	1002	6	Pisum sativum	Light responsive element

Gene name	*Cis*-acting elements name	Core sequence	Position	Length	Species	Type
LcNCED1	G-Box	CACGTG	1564	6	Pisum sativum	Light responsive element
LcNCED1	G-Box	CACGTG	1804	6	Pisum sativum	Light responsive element
LcNCED1	ATC-motif	AGTAATCT	576	8	Spinacia oleracea	Light responsive element
LcNCED1	Box 4	ATTAAT	406	6	Petroselinum crispum	Light responsive element
LcNCED1	Box 4	ATTAAT	625	6	Petroselinum crispum	Light responsive element
LcNCED1	Box 4	ATTAAT	736	6	Petroselinum crispum	Light responsive element
LcNCED1	Box 4	ATTAAT	959	6	Petroselinum crispum	Light responsive element
LcNCED1	Box 4	ATTAAT	986	6	Petroselinum crispum	Light responsive element
LcNCED1	Box 4	ATTAAT	1178	6	Petroselinum crispum	Light responsive element
LcNCED1	GATA-motif	AAGATAAGATT	1266	10	Arabidopsis thaliana	Light responsive element
LcNCED1	LAMP-element	CTTTATCA	1364	8	Pisum sativum	Light responsive element
LcNCED1	TCT-motif	TCTTAC	477	6	Arabidopsis thaliana	Light responsive element
LcNCED1	TCT-motif	TCTTAC	1816	6	Arabidopsis thaliana	Light responsive element
LcNCED1	AT1-motif	AATTATTTTTTATT	10	13	Solanum tuberosum	Light responsive element
LcNCED2	G-Box	CACGTG	1571	6	Pisum sativum	Light responsive element
LcNCED2	G-Box	CACGTG	1771	6	Pisum sativum	Light responsive element
LcNCED2	G-box	GCCACGTGGA	1569	10	Arabidopsis thaliana	Light responsive element
LcNCED2	G-box	CACGTG	1571	6	Arabidopsis thaliana	Light responsive element
LcNCED2	G-box	CACGTG	1771	6	Arabidopsis thaliana	Light responsive element

Gene name	*Cis*-acting elements name	Core sequence	Position	Length	Species	Type
LcNCED2	GT1-motif	GGTTAAT	792	7	Avena sativa	Light responsive element
LcNCED2	GT1-motif	GGTTAA	793	6	Arabidopsis thaliana	Light responsive element
LcNCED2	GT1-motif	GGTTAA	1688	6	Arabidopsis thaliana	Light responsive element
LcNCED2	Sp1	GGGCGG	1659	6	Oryza sativa	Light responsive element
LcNCED2	ATC-motif	AGCTATCCA	1575	9	Arabidopsis thaliana	Light responsive element
LcNCED2	Box 4	ATTAAT	764	6	Petroselinum crispum	Light responsive element
LcNCED2	Box 4	ATTAAT	992	6	Petroselinum crispum	Light responsive element
LcNCED2	Box 4	ATTAAT	1211	6	Petroselinum crispum	Light responsive element
LcNCED2	Box II	CCACGTGGC	1569	9	Petroselinum crispum	Light responsive element
LcNCED2	GATA-motif	AAGATAAGATT	1029	10	Arabidopsis thaliana	Light responsive element
LcNCED3	G-box	CACGTC	426	6	Zea mays	Light responsive element
LcNCED3	G-box	TACGTG	1169	6	Arabidopsis thaliana	Light responsive element
LcNCED3	G-box	CACGAC	1684	6	Zea mays	Light responsive element
LcNCED3	G-box	CACGTG	1813	6	Arabidopsis thaliana	Light responsive element
LcNCED3	G-Box	CACGTT	448	6	Pisum sativum	Light responsive element
LcNCED3	G-Box	CACGTT	956	6	Pisum sativum	Light responsive element
LcNCED3	G-Box	CACGTG	1813	6	Pisum sativum	Light responsive element
LcNCED3	ATCT-motif	AATCTAATCC	1539	9	Pisum sativum	Light responsive element
LcNCED3	Box 4	ATTAAT	268	6	Petroselinum crispum	Light responsive element

Gene name	*Cis*-acting elements name	Core sequence	Position	Length	Species	Type
LcNCED3	Box 4	ATTAAT	544	6	Petroselinum crispum	Light responsive element
LcNCED3	Box 4	ATTAAT	622	6	Petroselinum crispum	Light responsive element
LcNCED3	Box 4	ATTAAT	810	6	Petroselinum crispum	Light responsive element
LcNCED3	GATA-motif	GATAGGG	148	7	Pisum sativum	Light responsive element
LcNCED3	GATA-motif	GATAGGA	247	7	Arabidopsis thaliana	Light responsive element
LcNCED3	TCT-motif	TCTTAC	1481	6	Arabidopsis thaliana	Light responsive element
LcCCD1	ABRE	ACGTG	460	5	Arabidopsis thaliana	Abscisic acid responsive element
LcCCD1	ABRE	ACGTG	1680	5	Arabidopsis thaliana	Abscisic acid responsive element
LcCCD1	ABRE	CACGTG	1912	6	Arabidopsis thaliana	Abscisic acid responsive element
LcCCD1	ABRE	ACGTG	1913	5	Arabidopsis thaliana	Abscisic acid responsive element
LcCCD1	ABRE	ACGTG	1984	5	Arabidopsis thaliana	Abscisic acid responsive element
LcCCDlike-b	ABRE	ACGTG	668	5	Arabidopsis thaliana	Abscisic acid responsive element
LcCCDlike-b	ABRE	ACGTG	811	5	Arabidopsis thaliana	Abscisic acid responsive element
LcCCDlike-b	ABRE	ACGTG	1081	5	Arabidopsis thaliana	Abscisic acid responsive element
LcCCDlike-b	ABRE	CGTACGTGCA	1323	9	Hordeum vulgare	Abscisic acid responsive element
LcCCDlike-b	ABRE	ACGTG	1325	5	Arabidopsis thaliana	Abscisic acid responsive element
LcCCDlike-b	ABRE	ACGTG	1533	5	Arabidopsis thaliana	Abscisic acid responsive element
LcCCDlike-b	ABRE	ACGTG	1771	5	Arabidopsis thaliana	Abscisic acid responsive element
LcCCD4	ABRE	ACGTG	1620	5	Arabidopsis thaliana	Abscisic acid responsive element

Gene name	*Cis*-acting elements name	Core sequence	Position	Length	Species	Type
LcCCD4	ABRE	ACGTG	1844	5	Arabidopsis thaliana	Abscisic acid responsive element
LcCCD4b	ABRE	ACGTG	109	5	Arabidopsis thaliana	Abscisic acid responsive element
LcCCD4c1	ABRE	CGCACGTGTC	1374	9	Hordeum vulgare	Abscisic acid responsive element
LcCCD4c1	ABRE	CACGTG	1376	6	Arabidopsis thaliana	Abscisic acid responsive element
LcCCD4c1	ABRE	ACGTG	1377	5	Arabidopsis thaliana	Abscisic acid responsive element
LcCCD4c1	ABRE	ACGTG	1475	5	Arabidopsis thaliana	Abscisic acid responsive element
LcCCD4c1	ABRE	ACGTG	1613	5	Arabidopsis thaliana	Abscisic acid responsive element
LcCCD4c2	ABRE	ACGTG	800	5	Arabidopsis thaliana	Abscisic acid responsive element
LcCCD7	ABRE	ACGTG	1213	5	Arabidopsis thaliana	Abscisic acid responsive element
LcCCD7	ABRE	ACGTG	1828	5	Arabidopsis thaliana	Abscisic acid responsive element
LcCCD8b	ABRE	ACGTG	188	5	Arabidopsis thaliana	Abscisic acid responsive element
LcNCED1	ABRE	ACGTG	1002	5	Arabidopsis thaliana	Abscisic acid responsive element
LcNCED1	ABRE	GACACGTGGC	1562	9	Triticum aestivum	Abscisic acid responsive element
LcNCED1	ABRE	CACGTG	1564	6	Arabidopsis thaliana	Abscisic acid responsive element
LcNCED1	ABRE	ACGTG	1565	5	Arabidopsis thaliana	Abscisic acid responsive element
LcNCED1	ABRE	ACGTG	1610	5	Arabidopsis thaliana	Abscisic acid responsive element
LcNCED1	ABRE	CACGTG	1804	6	Arabidopsis thaliana	Abscisic acid responsive element
LcNCED1	ABRE	ACGTG	1805	5	Arabidopsis thaliana	Abscisic acid responsive element
LcNCED2	ABRE	CACGTG	1571	6	Arabidopsis thaliana	Abscisic acid responsive element

Gene name	*Cis*-acting elements name	Core sequence	Position	Length	Species	Type
LcNCED2	ABRE	ACGTG	1572	5	Arabidopsis thaliana	Abscisic acid responsive element
LcNCED2	ABRE	CACGTG	1771	6	Arabidopsis thaliana	Abscisic acid responsive element
LcNCED2	ABRE	ACGTG	1772	5	Arabidopsis thaliana	Abscisic acid responsive element
LcNCED3	ABRE	ACGTG	426	5	Arabidopsis thaliana	Abscisic acid responsive element
LcNCED3	ABRE	ACGTG	448	5	Arabidopsis thaliana	Abscisic acid responsive element
LcNCED3	ABRE	ACGTG	956	5	Arabidopsis thaliana	Abscisic acid responsive element
LcNCED3	ABRE	GACACGTACGT	1163	10	Oryza sativa	Abscisic acid responsive element
LcNCED3	ABRE	ACGTG	1170	5	Arabidopsis thaliana	Abscisic acid responsive element
LcNCED3	ABRE	CACGTG	1813	6	Arabidopsis thaliana	Abscisic acid responsive element
LcNCED3	ABRE	ACGTG	1814	5	Arabidopsis thaliana	Abscisic acid responsive element
LcCCD1	TGA-element	AACGAC	756	6	Brassica oleracea	Auxin responsive element
LcCCDlike-b	TGA-element	AACGAC	238	6	Brassica oleracea	Auxin responsive element
LcCCDlike-b	AuxRR-core	GGTCCAT	283	7	Nicotiana tabacum	Auxin responsive element
LcCCD4c1	TGA-element	AACGAC	1289	6	Brassica oleracea	Auxin responsive element
LcCCD4c1	AuxRR-core	GGTCCAT	1581	7	Nicotiana tabacum	Auxin responsive element
LcCCD4c2	TGA-element	AACGAC	387	6	Brassica oleracea	Auxin responsive element
LcCCD7	TGA-element	AACGAC	1229	6	Brassica oleracea	Auxin responsive element
LcCCD8b	AuxRR-core	GGTCCAT	155	7	Nicotiana tabacum	Auxin responsive element
LcCCD1	G-Box	CACGTT	1680	6	Pisum sativum	Gibberellin responsive element

Gene name	*Cis*-acting elements name	Core sequence	Position	Length	Species	Type
LcCCD1	TCA-element	CCATCTTTTT	1383	9	Nicotiana tabacum	Gibberellin responsive element
LcCCDlike-a	GARE-motif	TCTGTTG	502	7	Brassica oleracea	Gibberellin responsive element
LcCCDlike-a	TATC-box	TATCCCA	589	7	Oryza sativa	Gibberellin responsive element
LcCCDlike-b	GARE-motif	TCTGTTG	1765	7	Brassica oleracea	Gibberellin responsive element
LcCCD4a1	P-box	CCTTTTG	701	7	Oryza sativa	Gibberellin responsive element
LcCCD4a2	P-box	CCTTTTG	651	7	Oryza sativa	Gibberellin responsive element
LcCCD4b	TATC-box	TATCCCA	1040	7	Oryza sativa	Gibberellin responsive element
LcCCD4c1	TATC-box	TATCCCA	560	7	Oryza sativa	Gibberellin responsive element
LcCCD4c1	GARE-motif	TCTGTTG	1518	7	Brassica oleracea	Gibberellin responsive element
LcCCD4c1	P-box	CCTTTTG	978	7	Oryza sativa	Gibberellin responsive element
LcCCD4c2	GARE-motif	TCTGTTG	1226	7	Brassica oleracea	Gibberellin responsive element
LcCCD7	GARE-motif	TCTGTTG	754	7	Brassica oleracea	Gibberellin responsive element
LcNCED2	TATC-box	TATCCCA	1374	7	Oryza sativa	Gibberellin responsive element
LcNCED2	P-box	CCTTTTG	1590	7	Oryza sativa	Gibberellin responsive element
LcNCED3	P-box	CCTTTTG	553	7	Oryza sativa	Gibberellin responsive element
LcCCD1	TGACG-motif	TGACG	1727	5	Hordeum vulgare	MeJA responsive element
LcCCD1	GARE-motif	TCTGTTG	1105	7	Brassica oleracea	MeJA responsive element
LcCCDlike-b	CGTCA-motif	CGTCA	428	5	Hordeum vulgare	MeJA responsive element
LcCCDlike-b	CGTCA-motif	CGTCA	666	5	Hordeum vulgare	MeJA responsive element

Gene name	*Cis*-acting elements name	Core sequence	Position	Length	Species	Type
LcCCDlike-b	TGACG-motif	TGACG	428	5	Hordeum vulgare	MeJA responsive element
LcCCDlike-b	TGACG-motif	TGACG	666	5	Hordeum vulgare	MeJA responsive element
LcCCD4	CGTCA-motif	CGTCA	370	5	Hordeum vulgare	MeJA responsive element
LcCCD4	TGACG-motif	TGACG	370	5	Hordeum vulgare	MeJA responsive element
LcCCD4a1	CGTCA-motif	CGTCA	393	5	Hordeum vulgare	MeJA responsive element
LcCCD4a1	CGTCA-motif	CGTCA	1516	5	Hordeum vulgare	MeJA responsive element
LcCCD4a1	TGACG-motif	TGACG	393	5	Hordeum vulgare	MeJA responsive element
LcCCD4a1	TGACG-motif	TGACG	1516	5	Hordeum vulgare	MeJA responsive element
LcCCD4a2	CGTCA-motif	CGTCA	569	5	Hordeum vulgare	MeJA responsive element
LcCCD4a2	TGACG-motif	TGACG	569	5	Hordeum vulgare	MeJA responsive element
LcCCD4b	CGTCA-motif	CGTCA	1455	5	Hordeum vulgare	MeJA responsive element
LcCCD4b	CGTCA-motif	CGTCA	1846	5	Hordeum vulgare	MeJA responsive element
LcCCD4b	TGACG-motif	TGACG	1455	5	Hordeum vulgare	MeJA responsive element
LcCCD4b	TGACG-motif	TGACG	1846	5	Hordeum vulgare	MeJA responsive element
LcCCD4c2	CGTCA-motif	CGTCA	798	5	Hordeum vulgare	MeJA responsive element
LcCCD4c2	TGACG-motif	TGACG	798	5	Hordeum vulgare	MeJA responsive element
LcCCD7	CGTCA-motif	CGTCA	1024	5	Hordeum vulgare	MeJA responsive element
LcCCD7	TGACG-motif	TGACG	1024	5	Hordeum vulgare	MeJA responsive element
LcNCED1	CGTCA-motif	CGTCA	1608	5	Hordeum vulgare	MeJA responsive element

Gene name	*Cis*-acting elements name	Core sequence	Position	Length	Species	Type
LcNCED1	TGACG-motif	TGACG	1608	5	Hordeum vulgare	MeJA responsive element
LcNCED2	CGTCA-motif	CGTCA	1547	5	Hordeum vulgare	MeJA responsive element
LcNCED2	TGACG-motif	TGACG	1547	5	Hordeum vulgare	MeJA responsive element
LcNCED3	CGTCA-motif	CGTCA	428	5	Hordeum vulgare	MeJA responsive element
LcNCED3	CGTCA-motif	CGTCA	1100	5	Hordeum vulgare	MeJA responsive element
LcNCED3	CGTCA-motif	CGTCA	1103	5	Hordeum vulgare	MeJA responsive element
LcNCED3	TGACG-motif	TGACG	428	5	Hordeum vulgare	MeJA responsive element
LcNCED3	TGACG-motif	TGACG	1100	5	Hordeum vulgare	MeJA responsive element
LcNCED3	TGACG-motif	TGACG	1103	5	Hordeum vulgare	MeJA responsive element
LcCCD1	TCT-motif	TCTTAC	797	6	Arabidopsis thaliana	Salicylic acid responsive element
LcCCD4a2	TCA-element	TCAGAAGAGG	351	9	Brassica oleracea	Salicylic acid responsive element
LcCCD8a	TCA-element	CCATCTTTTT	862	9	Nicotiana tabacum	Salicylic acid responsive element
LcNCED1	TCA-element	CCATCTTTTT	1473	9	Nicotiana tabacum	Salicylic acid responsive element
LcNCED2	TCA-element	CCATCTTTTT	474	9	Nicotiana tabacum	Salicylic acid responsive element
LcCCD1	ARE	AAACCA	560	6	Zea mays	Anaerobic induction element
LcCCD1	ARE	AAACCA	931	6	Zea mays	Anaerobic induction element
LcCCD1	ARE	AAACCA	1225	6	Zea mays	Anaerobic induction element
LcCCD1	ATC-motif	AGTAATCT	1109	8	Spinacia oleracea	Anaerobic induction element
LcCCDlike-a	ARE	AAACCA	388	6	Zea mays	Anaerobic induction element

Gene name	*Cis*-acting elements name	Core sequence	Position	Length	Species	Type
LcCCDlike-a	ARE	AAACCA	1301	6	Zea mays	Anaerobic induction element
LcCCDlike-a	ARE	AAACCA	1572	6	Zea mays	Anaerobic induction element
LcCCDlike-a	ARE	AAACCA	1662	6	Zea mays	Anaerobic induction element
LcCCD4	ARE	AAACCA	1245	6	Zea mays	Anaerobic induction element
LcCCD4	ARE	AAACCA	1381	6	Zea mays	Anaerobic induction element
LcCCD4	ARE	AAACCA	1730	6	Zea mays	Anaerobic induction element
LcCCD4a1	ARE	AAACCA	172	6	Zea mays	Anaerobic induction element
LcCCD4a1	ARE	AAACCA	193	6	Zea mays	Anaerobic induction element
LcCCD4a1	ARE	AAACCA	323	6	Zea mays	Anaerobic induction element
LcCCD4a1	GC-motif	CCCCCG	1723	6	Zea mays	Anaerobic induction element
LcCCD4a2	ARE	AAACCA	280	6	Zea mays	Anaerobic induction element
LcCCD4a2	ARE	AAACCA	306	6	Zea mays	Anaerobic induction element
LcCCD4a2	GC-motif	CCCCCG	1340	6	Zea mays	Anaerobic induction element
LcCCD4b	ARE	AAACCA	936	6	Zea mays	Anaerobic induction element
LcCCD4c1	ARE	AAACCA	1735	6	Zea mays	Anaerobic induction element
LcCCD4c1	ARE	AAACCA	1852	6	Zea mays	Anaerobic induction element
LcCCD7	ARE	AAACCA	392	6	Zea mays	Anaerobic induction element
LcCCD7	ARE	AAACCA	1535	6	Zea mays	Anaerobic induction element
LcCCD7	ARE	AAACCA	1882	6	Zea mays	Anaerobic induction element

Gene name	*Cis*-acting elements name	Core sequence	Position	Length	Species	Type
LcCCD8b	ARE	AAACCA	711	6	Zea mays	Anaerobic induction element
LcNCED1	ARE	AAACCA	798	6	Zea mays	Anaerobic induction element
LcNCED1	ARE	AAACCA	1352	6	Zea mays	Anaerobic induction element
LcNCED2	ARE	AAACCA	1223	6	Zea mays	Anaerobic induction element
LcNCED2	ARE	AAACCA	1932	6	Zea mays	Anaerobic induction element
LcNCED2	ARE	AAACCA	1944	6	Zea mays	Anaerobic induction element
LcNCED3	ARE	AAACCA	1335	6	Zea mays	Anaerobic induction element
LcNCED3	ARE	AAACCA	1372	6	Zea mays	Anaerobic induction element
LcNCED3	ARE	AAACCA	1538	6	Zea mays	Anaerobic induction element
LcNCED3	ARE	AAACCA	1698	6	Zea mays	Anaerobic induction element
LcCCDlike-a	MBS	CAACTG	993	6	Arabidopsis thaliana	MYB binding site involved in drought-inducibility
LcCCDlike-a	MBS	CAACTG	1040	6	Arabidopsis thaliana	MYB binding site involved in drought-inducibility
LcCCDlike-a	MBS	CAACTG	1054	6	Arabidopsis thaliana	MYB binding site involved in drought-inducibility
LcCCDlike-a	MBS	CAACTG	1247	6	Arabidopsis thaliana	MYB binding site involved in drought-inducibility
LcCCDlike-b	MBS	CAACTG	1401	6	Arabidopsis thaliana	MYB binding site involved in drought-inducibility
LcCCDlike-b	MBS	CAACTG	1455	6	Arabidopsis thaliana	MYB binding site involved in drought-inducibility
LcCCDlike-b	MBS	CAACTG	1982	6	Arabidopsis thaliana	MYB binding site involved in drought-inducibility
LcCCD4	MBS	CAACTG	679	6	Arabidopsis thaliana	MYB binding site involved in drought-inducibility
LcCCD4a1	MBS	CAACTG	804	6	Arabidopsis thaliana	MYB binding site involved in drought-inducibility

Gene name	*Cis*-acting elements name	Core sequence	Position	Length	Species	Type
LcCCD4a2	MBS	CAACTG	752	6	Arabidopsis thaliana	MYB binding site involved in drought-inducibility
LcCCD4a2	MBS	CAACTG	1192	6	Arabidopsis thaliana	MYB binding site involved in drought-inducibility
LcCCD4c2	MBS	CAACTG	1988	6	Arabidopsis thaliana	MYB binding site involved in drought-inducibility
LcCCD8b	MBS	CAACTG	1747	6	Arabidopsis thaliana	MYB binding site involved in drought-inducibility
LcNCED1	MBS	CAACTG	319	6	Arabidopsis thaliana	MYB binding site involved in drought-inducibility
LcNCED2	MBS	CAACTG	1319	6	Arabidopsis thaliana	MYB binding site involved in drought-inducibility
LcCCD1	MRE	AACCTAA	1031	7	Petroselinum crispum	Low-temperature responsive element
LcCCDlike-b	LTR	CCGAAA	1636	6	Hordeum vulgare	Low-temperature responsive element
LcCCD4a1	LTR	CCGAAA	261	6	Hordeum vulgare	Low-temperature responsive element
LcCCD4a1	LTR	CCGAAA	1396	6	Hordeum vulgare	Low-temperature responsive element
LcCCD4a2	LTR	CCGAAA	218	6	Hordeum vulgare	Low-temperature responsive element
LcCCD4c2	LTR	CCGAAA	255	6	Hordeum vulgare	Low-temperature responsive element
LcCCD4c2	LTR	CCGAAA	544	6	Hordeum vulgare	Low-temperature responsive element
LcCCD4c2	LTR	CCGAAA	641	6	Hordeum vulgare	Low-temperature responsive element
LcCCD4c2	LTR	CCGAAA	738	6	Hordeum vulgare	Low-temperature responsive element
LcCCD7	LTR	CCGAAA	682	6	Hordeum vulgare	Low-temperature responsive element
LcCCD8b	LTR	CCGAAA	67	6	Hordeum vulgare	Low-temperature responsive element
LcNCED2	LTR	CCGAAA	1610	6	Hordeum vulgare	Low-temperature responsive element
LcCCDlike-b	TC-rich repeats	ATTCTCTAAC	1795	9	Nicotiana tabacum	Defense and stress-responsive element

Gene name	*Cis*-acting elements name	Core sequence	Position	Length	Species	Type
LcCCD4b	TC-rich repeats	GTTTTCTTAC	231	9	Nicotiana tabacum	Defense and stress-responsive element
LcCCD4c1	TC-rich repeats	GTTTTCTTAC	839	9	Nicotiana tabacum	Defense and stress-responsive element
LcCCD4c1	TC-rich repeats	GTTTTCTTAC	898	9	Nicotiana tabacum	Defense and stress-responsive element
LcCCD8a	TC-rich repeats	ATTCTCTAAC	1502	9	Nicotiana tabacum	Defense and stress-responsive element
LcNCED1	TC-rich repeats	GTTTTCTTAC	477	9	Nicotiana tabacum	Defense and stress-responsive element
LcNCED2	TC-rich repeats	GTTTTCTTAC	523	9	Nicotiana tabacum	Defense and stress-responsive element
LcNCED3	TC-rich repeats	GTTTTCTTAC	1477	9	Nicotiana tabacum	Defense and stress-responsive element
LcNCED3	TC-rich repeats	GTTTTCTTAC	1781	9	Nicotiana tabacum	Defense and stress-responsive element
LcCCD4c2	MBSI	aaaAaaC(G/C)GTTA	50	10.5	Petunia hybrida	MYB binding site involved in flavonoid biosynthetic genes regulation
LcCCD8b	MBSI	TTTTTACGGTTA	1642	11	Petunia hybrida	MYB binding site involved in flavonoid biosynthetic genes regulation
LcCCDlike-a	CAT-box	GCCACT	257	6	Arabidopsis thaliana	Meristem expression element
LcCCD4	CAT-box	GCCACT	496	6	Arabidopsis thaliana	Meristem expression element
LcCCD4b	CAT-box	GCCACT	470	6	Arabidopsis thaliana	Meristem expression element
LcCCD4b	CAT-box	GCCACT	1841	6	Arabidopsis thaliana	Meristem expression element
LcCCD4c1	CAT-box	GCCACT	1397	6	Arabidopsis thaliana	Meristem expression element
LcCCD8a	CAT-box	GCCACT	699	6	Arabidopsis thaliana	Meristem expression element
LcCCD8b	CAT-box	GCCACT	108	6	Arabidopsis thaliana	Meristem expression element
LcNCED2	CAT-box	GCCACT	498	6	Arabidopsis thaliana	Meristem expression element
LcNCED3	CAT-box	GCCACT	978	6	Arabidopsis thaliana	Meristem expression element

Gene name	*Cis*-acting elements name	Core sequence	Position	Length	Species	Type
LcNCED3	CAT-box	GCCACT	1742	6	Arabidopsis thaliana	Meristem expression element
LcCCD1	GT1-motif	GTGTGTGAA	446	9	Solanum tuberosum	Endosperm expression element
LcCCDlike-a	GCN4_motif	TGAGTCA	1400	7	Oryza sativa	Endosperm expression element
LcCCD4	GCN4_motif	TGAGTCA	1386	7	Oryza sativa	Endosperm expression element
LcCCD7	GCN4_motif	TGAGTCA	841	7	Oryza sativa	Endosperm expression element
LcCCDlike-a	circadian	CAAAGATATC	399	9	Lycopersicon esculentum	Circadian responsive element
LcCCD4b	circadian	CAAAGATATC	36	9	Lycopersicon esculentum	Circadian responsive element
LcCCD4c1	circadian	CAAAGATATC	223	9	Lycopersicon esculentum	Circadian responsive element
LcNCED1	RY-element	CATGCATG	217	8	Helianthus annuus	Seed-specific regulatory element
LcCCDlike-b	O2-site	GATGA(C/T)(A/G)TG(A/G)	411	8	Zea mays	Zein metabolism regulatory element
LcCCD4	O2-site	GATGACATGG	1891	9	Zea mays	Zein metabolism regulatory element
LcCCD4a2	O2-site	GATGACATGG	335	9	Zea mays	Zein metabolism regulatory element
LcCCD7	O2-site	GATGACATGG	1538	9	Zea mays	Zein metabolism regulatory element
LcCCD8b	O2-site	GATGA(C/T)(A/G)TG(A/G)	1054	8.5	Zea mays	Zein metabolism regulatory element

Supplement Table S3. Two-dimensional structures of LcCCO proteins.

Gene name	Sequence length	Core Sequence Position Lenggh		
		Alpha helix (Hh)	Extended strand (Ee)	Random coil (Cc)
LcCCD1	1434	412/28.73%	302/21.06%	720/50.21%
LcCCDlike-a	1172	246/20.99%	267/22.78%	659/56.23%
LcCCDlike-b	359	37/10.31%	97/27.02%	225/62.67%
LcCCD4	589	104/17.66%	116/19.69%	369/62.65%
LcCCD4a1	584	75/12.84%	157/26.88%	352/60.27%
LcCCD4a2	584	77/13.18%	164/28.08%	343/58.73%
LcCCD4b	577	96/16.64%	131/22.70%	350/60.66%
LcCCD4c1	303	67/22.11%	68/22.44%	168/55.45%
LcCCD4c2	566	116/20.07%	132/22.84%	330/57.09%
LcCCD7	628	120/19.11%	153/24.36%	355/56.53%
LcCCD8a	548	118/21.53%	119/21.72%	311/56.75%
LcCCD8b	566	88/15.55%	139/24.56%	339/59.89%
LcNCED1	596	158/26.51%	97/16.28%	341/57.21%
LcNCED2	598	142/23.75%	121/20.23%	335/56.02%
LcNCED3	601	94/15.64%	140/23.29%	367/61.06%

Supplement Table S4. GO enrichment analysis of *LcCCO* genes.

Class	GO_Name	GO_ID	GO_Level	P_value	Enrichment Score	Hits Genes Counts In Selected Set	Hits Genes Counts In Background	All Genes Counts In Selected Set	All Genes Counts In Background	Genes Of Selected Set In GO term	corrected *p*-value (BH method)
**Molecular function**	catalytic activity	GO:0003824	2	8.29E-04	1.604673073	15	26535	15	42580	LITCHI012579.m1,LITCHI006417.m1,LITCHI015114.m1,LITCHI017175.m1,LITCHI015831.m1,LITCHI000409.m1,LITCHI000422.m1,LITCHI028785.m1,LITCHI017848.m1,LITCHI015832.m1,LITCHI000415.m1,LITCHI006516.m1,LITCHI001770.m1,LITCHI017183.m1,LITCHI004397.m1,	0.004973991
**Cellular component**	thylakoid	GO:0009579	4	0	19.02987619	13	2073	15	45518	LITCHI012579.m1,LITCHI006417.m1,LITCHI015114.m1,LITCHI017175.m1,LITCHI015831.m1,LITCHI000409.m1,LITCHI000422.m1,LITCHI028785.m1,LITCHI017848.m1,LITCHI015832.m1,LITCHI000415.m1,LITCHI001770.m1,LITCHI004397.m1,	0
**Cellular component**	chloroplast	GO:0009507	7	5.91E-10	4.121140788	15	11045	15	45518	LITCHI012579.m1,LITCHI006417.m1,LITCHI015114.m1,LITCHI017175.m1,LITCHI015831.m1,LITCHI000409.m1,LITCHI000422.m1,LITCHI028785.m1,LITCHI017848.m1,LITCHI015832.m1,LITCHI000415.m1,LITCHI006516.m1,LITCHI001770.m1,LITCHI017183.m1,LITCHI004397.m1,	3.84E-09
**Cellular component**	plastid	GO:0009536	6	8.56E-10	4.020669552	15	11321	15	45518	LITCHI012579.m1,LITCHI006417.m1,LITCHI015114.m1,LITCHI017175.m1,LITCHI015831.m1,LITCHI000409.m1,LITCHI000422.m1,LITCHI028785.m1,LITCHI017848.m1,LITCHI015832.m1,LITCHI000415.m1,LITCHI006516.m1,LITCHI001770.m1,LITCHI017183.m1,LITCHI004397.m1,	3.71E-09

Class	GO_Name	GO_ID	GO_Level	P_value	Enrichment Score	Hits Genes Counts In Selected Set	Hits Genes Counts In Background	All Genes Counts In Selected Set	All Genes Counts In Background	Genes Of Selected Set In GO term	corrected *p*-value (BH method)
**Cellular component**	Golgi apparatus	GO:0005794	6	1.81E-07	4.649438202	12	7832	15	45518	LITCHI012579.m1,LITCHI015114.m1,LITCHI017175.m1,LITCHI015831.m1,LITCHI000409.m1,LITCHI000422.m1,LITCHI028785.m1,LITCHI017848.m1,LITCHI015832.m1,LITCHI000415.m1,LITCHI001770.m1,LITCHI004397.m1,	5.89E-07
**Cellular component**	plasma membrane	GO:0005886	4	5.94E-07	2.600137096	15	17506	15	45518	LITCHI012579.m1,LITCHI006417.m1,LITCHI015114.m1,LITCHI017175.m1,LITCHI015831.m1,LITCHI000409.m1,LITCHI000422.m1,LITCHI028785.m1,LITCHI017848.m1,LITCHI015832.m1,LITCHI000415.m1,LITCHI006516.m1,LITCHI001770.m1,LITCHI017183.m1,LITCHI004397.m1,	1.54E-06
**Cellular component**	vacuole	GO:0005773	6	8.51E-07	4.059576366	12	8970	15	45518	LITCHI012579.m1,LITCHI015114.m1,LITCHI017175.m1,LITCHI015831.m1,LITCHI000409.m1,LITCHI000422.m1,LITCHI028785.m1,LITCHI017848.m1,LITCHI015832.m1,LITCHI000415.m1,LITCHI001770.m1,LITCHI004397.m1,	1.84E-06
**Cellular component**	membrane	GO:0016020	3	5.95E-04	1.64052476	15	27746	15	45518	LITCHI012579.m1,LITCHI006417.m1,LITCHI015114.m1,LITCHI017175.m1,LITCHI015831.m1,LITCHI000409.m1,LITCHI000422.m1,LITCHI028785.m1,LITCHI017848.m1,LITCHI015832.m1,LITCHI000415.m1,LITCHI006516.m1,LITCHI001770.m1,LITCHI017183.m1,LITCHI004397.m1,	0.001105253
**Biological process**	fruit ripening	GO:0009835	5	0	63.98003565	12	561	15	44866	LITCHI012579.m1,LITCHI015114.m1,LITCHI017175.m1,LITCHI015831.m1,LITCHI000409.m1,LITCHI000422.m1,LITCHI028785.m1,LITCHI017848.m1,LITCHI015832.m1,LITCHI000415.m1,LITCHI001770.m	0

Class	GO_Name	GO_ID	GO_Level	P_value	Enrichment Score	Hits Genes Counts In Selected Set	Hits Genes Counts In Background	All Genes Counts In Selected Set	All Genes Counts In Background	Genes Of Selected Set In GO term	corrected *p*-value (BH method)
										1,LITCHI004397.m1,	
**Biological process**	pollination	GO:0009856	3	3.87E-13	14.19810127	12	2528	15	44866	LITCHI012579.m1,LITCHI015114.m1,LITCHI017175.m1,LITCHI015831.m1,LITCHI000409.m1,LITCHI000422.m1,LITCHI028785.m1,LITCHI017848.m1,LITCHI015832.m1,LITCHI000415.m1,LITCHI001770.m1,LITCHI004397.m1,	4.65E-12
**Biological process**	lipid metabolic process	GO:0006629	4	4.32E-13	6.666567608	15	6730	15	44866	LITCHI012579.m1,LITCHI006417.m1,LITCHI015114.m1,LITCHI017175.m1,LITCHI015831.m1,LITCHI000409.m1,LITCHI000422.m1,LITCHI028785.m1,LITCHI017848.m1,LITCHI015832.m1,LITCHI000415.m1,LITCHI006516.m1,LITCHI001770.m1,LITCHI017183.m1,LITCHI004397.m1,	3.45E-12
**Biological process**	flower development	GO:0009908	7	1.88E-09	6.90378919	12	5199	15	44866	LITCHI012579.m1,LITCHI015114.m1,LITCHI017175.m1,LITCHI015831.m1,LITCHI000409.m1,LITCHI000422.m1,LITCHI028785.m1,LITCHI017848.m1,LITCHI015832.m1,LITCHI000415.m1,LITCHI001770.m1,LITCHI004397.m1,	1.13E-08
**Biological process**	catabolic process	GO:0009056	3	3.21E-09	3.681765961	15	12186	15	44866	LITCHI012579.m1,LITCHI006417.m1,LITCHI015114.m1,LITCHI017175.m1,LITCHI015831.m1,LITCHI000409.m1,LITCHI000422.m1,LITCHI028785.m1,LITCHI017848.m1,LITCHI015832.m1,LITCHI000415.m1,LITCHI006516.m1,LITCHI001770.m1,LITCHI017183.m1,LITCHI004397.m1,	1.54E-08
**Biological process**	response to endogenous	GO:0009719	3	3.04E-08	3.169622042	15	14155	15	44866	LITCHI012579.m1,LITCHI006417.m1,LITCHI015114.m1,LITCHI017175.m1,LITCHI015831.m1,LITCHI000409.m1,LITCHI000422.m1,LITC	1.22E-07

Class	GO_Name	GO_ID	GO_Level	P_value	Enrichment Score	Hits Genes Counts In Selected Set	Hits Genes Counts In Background	All Genes Counts In Selected Set	All Genes Counts In Background	Genes Of Selected Set In GO term	corrected *p*-value (BH method)
	stimulus									HI028785.m1,LITCHI017848.m1,LITCHI015832.m1,LITCHI000415.m1,LITCHI006516.m1,LITCHI001770.m1,LITCHI017183.m1,LITCHI004397.m1,	
**Biological process**	signal transduction	GO:0007165	5	7.92E-08	2.973621421	15	15088	15	44866	LITCHI012579.m1,LITCHI006417.m1,LITCHI015114.m1,LITCHI017175.m1,LITCHI015831.m1,LITCHI000409.m1,LITCHI000422.m1,LITCHI028785.m1,LITCHI017848.m1,LITCHI015832.m1,LITCHI000415.m1,LITCHI006516.m1,LITCHI001770.m1,LITCHI017183.m1,LITCHI004397.m1,	2.72E-07
**Biological process**	cell communication	GO:0007154	3	5.27E-07	2.62067757	15	17120	15	44866	LITCHI012579.m1,LITCHI006417.m1,LITCHI015114.m1,LITCHI017175.m1,LITCHI015831.m1,LITCHI000409.m1,LITCHI000422.m1,LITCHI028785.m1,LITCHI017848.m1,LITCHI015832.m1,LITCHI000415.m1,LITCHI006516.m1,LITCHI001770.m1,LITCHI017183.m1,LITCHI004397.m1,	1.58E-06
**Biological process**	response to abiotic stimulus	GO:0009628	3	5.71E-07	2.60697269	15	17210	15	44866	LITCHI012579.m1,LITCHI006417.m1,LITCHI015114.m1,LITCHI017175.m1,LITCHI015831.m1,LITCHI000409.m1,LITCHI000422.m1,LITCHI028785.m1,LITCHI017848.m1,LITCHI015832.m1,LITCHI000415.m1,LITCHI006516.m1,LITCHI001770.m1,LITCHI017183.m1,LITCHI004397.m1,	1.52E-06
**Biological process**	response to light stimulus	GO:0009416	5	1.50E-05	4.167572338	10	7177	15	44866	LITCHI012579.m1,LITCHI006417.m1,LITCHI015114.m1,LITCHI000409.m1,LITCHI000422.m1,LITCHI028785.m1,LITCHI000415.m1,LITCHI006516.m1,LITCHI017183.m1,LITCHI004397.m1,	3.60E-05

Class	GO_Name	GO_ID	GO_Level	P_value	Enrichment Score	Hits Genes Counts In Selected Set	Hits Genes Counts In Background	All Genes Counts In Selected Set	All Genes Counts In Background	Genes Of Selected Set In GO term	corrected *p*-value (BH method)
**Biological process**	biosynthetic process	GO:0009058	3	3.09E-05	1.998129509	15	22454	15	44866	LITCHI012579.m1,LITCHI006417.m1,LITCHI015114.m1,LITCHI017175.m1,LITCHI015831.m1,LITCHI000409.m1,LITCHI000422.m1,LITCHI028785.m1,LITCHI017848.m1,LITCHI015832.m1,LITCHI000415.m1,LITCHI006516.m1,LITCHI001770.m1,LITCHI017183.m1,LITCHI004397.m1,	6.74E-05
**Biological process**	response to chemical	GO:0042221	3	3.76E-05	1.971958509	15	22752	15	44866	LITCHI012579.m1,LITCHI006417.m1,LITCHI015114.m1,LITCHI017175.m1,LITCHI015831.m1,LITCHI000409.m1,LITCHI000422.m1,LITCHI028785.m1,LITCHI017848.m1,LITCHI015832.m1,LITCHI000415.m1,LITCHI006516.m1,LITCHI001770.m1,LITCHI017183.m1,LITCHI004397.m1,	7.53E-05
**Biological process**	multicellular organism development	GO:0007275	4	5.96E-05	1.912365202	15	23461	15	44866	LITCHI012579.m1,LITCHI006417.m1,LITCHI015114.m1,LITCHI017175.m1,LITCHI015831.m1,LITCHI000409.m1,LITCHI000422.m1,LITCHI028785.m1,LITCHI017848.m1,LITCHI015832.m1,LITCHI000415.m1,LITCHI006516.m1,LITCHI001770.m1,LITCHI017183.m1,LITCHI004397.m1,	1.10E-04
**Biological process**	post-embryonic development	GO:0009791	3	1.10E-04	2.621060318	12	13694	15	44866	LITCHI012579.m1,LITCHI015114.m1,LITCHI017175.m1,LITCHI015831.m1,LITCHI000409.m1,LITCHI000422.m1,LITCHI028785.m1,LITCHI017848.m1,LITCHI015832.m1,LITCHI000415.m1,LITCHI001770.m1,LITCHI004397.m1,	1.89E-04
**Biological process**	anatomical structure	GO:0048856	3	2.31E-04	1.747186417	15	25679	15	44866	LITCHI012579.m1,LITCHI006417.m1,LITCHI015114.m1,LITCHI017175.m1,LITCHI015831.m1,LITCHI000409.m1,LITCHI000422.m1,LITC	3.70E-04

Class	GO_Name	GO_ID	GO_Level	P_value	Enrichment Score	Hits Genes Counts In Selected Set	Hits Genes Counts In Background	All Genes Counts In Selected Set	All Genes Counts In Background	Genes Of Selected Set In GO term	corrected *p*-value (BH method)
	development									HI028785.m1,LITCHI017848.m1,LITCHI015832.m1,LITCHI000415.m1,LITCHI006516.m1,LITCHI001770.m1,LITCHI017183.m1,LITCHI004397.m1,	
**Biological process**	reproduction	GO:0000003	2	9.12E-04	2.149526889	12	16698	15	44866	LITCHI012579.m1,LITCHI015114.m1,LITCHI017175.m1,LITCHI015831.m1,LITCHI000409.m1,LITCHI000422.m1,LITCHI028785.m1,LITCHI017848.m1,LITCHI015832.m1,LITCHI000415.m1,LITCHI001770.m1,LITCHI004397.m1,	0.001367911
**Biological process**	response to stress	GO:0006950	3	0.037819746	1.473069031	12	24366	15	44866	LITCHI012579.m1,LITCHI015114.m1,LITCHI017175.m1,LITCHI015831.m1,LITCHI000409.m1,LITCHI000422.m1,LITCHI028785.m1,LITCHI017848.m1,LITCHI015832.m1,LITCHI000415.m1,LITCHI001770.m1,LITCHI004397.m1,	0.050426328

Supplement Table S5. The TPM value and differential expression analysis of *LcCCO* genes during pericarp coloring of 'Feizixiao' litchi treated by exogenous CPPU.

Gene name	TPM value												log2(FoldChange)				P Value			
	CK1_1	CK1_2	CK1_3	CK2_1	CK2_2	CK2_3	T1_1	T1_2	T1_3	T2_1	T2_2	T2_3	CK1-vs-CK2	CK1-vs-T1	CK2-vs-T2	T1-vs-T2	CK1-vs-CK2	CK1-vs-T1	CK2-vs-T2	T1-vs-T2
LcCCD1	59.30	69.41	65.84	47.25	35.91	47.59	58.22	65.55	65.55	45.69	44.50	52.30	-0.92	-0.08	0.17	-0.67	0.18	0.91	0.80	0.29
LcCCDlike-a	0.44	0.21	0.26	0.15	0.15	0.48	0.13	0.07	0.08	0.41	0.09	0.35	0.05	-0.48	0.44	0.97	1.00	0.75	0.73	0.42
LcCCDlike-b	0.00	0.19	0.00	5.30	2.90	6.05	0.00	0.05	0.00	0.30	0.47	1.93	-0.19	-0.07	-2.35	-2.46	0.87	0.96	0.05	0.04
LcCCD4	36.19	88.04	52.46	134.03	111.64	83.05	50.16	95.99	43.11	54.00	50.52	76.84	0.50	0.05	-0.86	-0.41	0.48	0.95	0.21	0.55
LcCCD4a1	0.04	0.00	0.00	0.00	0.00	0.00	0.00	0.00	0.00	0.00	0.00	0.08	-0.26	-0.26	0.49	0.49	1.00	1.00	1.00	1.00
LcCCD4a2	0.00	0.02	0.00	0.00	0.00	0.00	0.00	0.02	0.00	0.00	0.00	0.00	-0.26	-0.26	0.14	0.14	1.00	1.00	1.00	1.00
LcCCD4b	0.00	0.00	0.00	0.00	0.00	0.00	0.00	0.03	0.00	0.00	0.00	0.00	-0.26	-0.26	0.14	0.14	1.00	1.00	1.00	1.00
LcCCD4c1	0.23	0.29	0.10	0.00	0.06	0.06	0.04	0.05	0.00	0.07	0.00	0.00	-1.77	-1.77	0.14	0.14	0.27	0.28	1.00	1.00
LcCCD4c2	0.12	0.07	0.02	0.00	0.00	0.00	0.17	0.00	0.02	0.00	0.00	0.00	-2.22	-0.16	0.14	-1.92	0.10	1.00	1.00	0.18
LcCCD7	0.00	0.00	0.00	0.00	0.00	0.00	0.00	0.00	0.00	0.00	0.00	0.00	-0.26	-0.26	0.14	0.14	1.00	1.00	1.00	1.00
LcCCD8a	0.15	1.62	0.35	0.70	0.25	0.37	0.14	0.25	0.23	0.69	0.69	0.83	-4.97	-4.97	0.14	0.14	0.01	0.01	1.00	1.00
LcCCD8b	0.00	0.00	0.00	0.00	0.00	0.00	0.00	0.00	0.00	0.00	0.00	0.00	-0.26	0.22	0.14	-0.35	1.00	1.00	1.00	1.00
LcNCED1	6.54	5.84	1.10	0.77	0.58	1.13	4.39	1.14	1.77	1.48	1.04	0.54	-2.91	-0.90	0.41	-1.59	0.02	0.42	0.71	0.16
LcNCED2	20.87	17.48	21.14	3.17	3.08	3.28	32.55	18.06	17.94	4.22	4.69	3.38	-3.01	0.15	0.35	-2.81	0.00	0.85	0.74	0.00
LcNCED3	0.02	0.03	0.00	0.02	0.00	0.07	0.00	0.00	0.00	0.00	0.00	0.02	0.09	-0.63	-0.58	0.14	1.00	1.00	0.76	1.00

Supplement Table S6. The TPM value and differential expression analysis of *LcCCO* genes of 'Feizixiao' litchi on the 0, 1, 3, and 7 days after bags removed (Zhang et al., 2016a).

Gene name	TPM value				log2 (FoldChange)			P Value		
	0 d	1 d	3 d	7 d	0 d-vs-1 d	0 d-vs-3 d	0 d-vs-7 d	0 d-vs-1 d	0 d-vs-3 d	0 d-vs-7 d
LcCCD1	10.80	14.12	11.80	14.07	0.42	0.08	0.45	0.00	0.35	0.00
LcCCDlike-a	0.00	0.00	0.06	0.04	-0.09	1.73	1.09	1.00	0.63	0.63
LcCCDlike-b	0.31	0.28	0.29	0.00	-0.16	-0.11	-2.58	1.00	1.00	0.22
LcCCD4	111.85	95.18	122.06	153.59	-0.20	0.09	0.51	0.00	0.04	0.00
LcCCD4a1	0.00	0.00	0.00	0.00	-0.09	0.32	-0.54	1.00	1.00	1.00
LcCCD4a2	0.04	0.00	0.00	0.00	-0.09	0.32	-0.54	1.00	1.00	1.00
LcCCD4b	0.00	0.00	0.00	0.00	-0.09	0.32	-0.54	1.00	1.00	1.00
LcCCD4c1	0.00	0.00	0.00	0.00	-0.09	0.32	-0.54	1.00	1.00	1.00
LcCCD4c2	0.00	0.00	0.00	0.00	-0.09	0.32	-0.54	1.00	1.00	1.00
LcCCD7	0.00	0.00	0.00	0.00	-0.09	0.32	-0.54	1.00	1.00	1.00
LcCCD8a	0.00	0.00	0.00	0.00	-0.09	0.32	-0.54	1.00	1.00	1.00
LcCCD8b	0.00	0.00	0.00	0.00	-0.09	0.32	-0.54	1.00	1.00	1.00
LcNCED1	24.73	15.27	49.47	114.86	-0.67	0.96	2.27	0.00	0.00	0.00
LcNCED2	18.71	12.18	34.77	33.81	-0.58	0.85	0.91	0.00	0.00	0.00
LcNCED3	0.04	0.10	0.20	0.08	1.46	2.34	1.09	0.63	0.38	0.63

Supplement Table S7. The TPM value and differential expression analysis of *LcCCO* genes in 'Nuomici' Litchi during three different development stages of fruit (Lai et al., 2015).

Gene name	TPM value			log2 (FoldChange)		P Value	
	Green	Yellow	Red	Green-vs-Yellow	Green-vs-Red	Green-vs-Yellow	Green-vs-Red
LcCCD1	100.27	53.77	63.34	-0.74	-0.51	0.00	0.00
LcCCDlike-a	0.22	0.00	0.09	-0.71	-1.15	1.00	1.00
LcCCDlike-b	0.00	0.00	0.00	0.29	-0.15	1.00	1.00
LcCCD4	210.19	177.69	142.03	-0.07	-0.41	0.36	0.00
LcCCD4a1	0.00	0.48	0.45	1.18	1.09	1.00	1.00
LcCCD4a2	0.00	0.04	2.22	-2.55	3.40	1.00	0.01
LcCCD4b	0.23	0.00	0.00	0.29	-0.15	1.00	1.00
LcCCD4c1	0.00	0.00	0.00	0.29	-0.15	1.00	1.00
LcCCD4c2	0.00	0.00	0.00	0.29	-0.15	1.00	1.00
LcCCD7	0.00	0.00	0.00	0.29	-0.15	1.00	1.00
LcCCD8a	0.00	0.00	0.00	0.29	-0.15	1.00	1.00
LcCCD8b	0.00	0.00	0.00	0.29	-0.15	1.00	1.00
LcNCED1	98.25	121.87	173.04	0.47	0.97	0.00	0.00

LcNCED2	**78.60**	**51.46**	**121.90**	**-0.45**	**0.79**	**0.00**	**0.00**
LcNCED3	**0.00**	**0.00**	**0.18**	**0.29**	**-0.15**	**1.00**	**1.00**

Supplement Table S8. The TPM value and differential expression analysis of *LcCCO* genes of the entire inflorescences samples of 'Feizixiao' litchi on 28 days after the uniconazole trea tment (Wei et al., 2017b).

Gene name	TPM value		log2(FoldChange)	P Value
	CK	T	T-vs-CK	CK-vs-T
LcCCD1	13.55	13.56	0.05	0.45
LcCCDlike-a	0.11	0.09	-0.17	1.00
LcCCDlike-b	0.67	1.09	0.75	0.27
LcCCD4	71.40	41.61	-0.74	0.00
LcCCD4a1	0.07	0.00	-0.94	1.00
LcCCD4a2	0.07	0.03	-0.94	1.00
LcCCD4b	0.00	0.00	0.00	1.00
LcCCD4c1	0.31	0.00	-1.97	0.38
LcCCD4c2	61.86	3.87	-3.94	0.00
LcCCD7	0.00	0.00	0.00	1.00
LcCCD8a	6.54	4.27	-0.59	0.00
LcCCD8b	0.87	0.28	-1.60	0.00
LcNCED1	19.24	17.54	-0.08	0.37
LcNCED2	89.87	32.09	-1.44	0.00
LcNCED3	166.32	52.37	-1.61	0.00

Supplement Table S9. The TPM value and differential expression analysis of *LcCCO* genes of fruit samples of 'Wuye' litchi after 2, 4, and 7 days treated by girdling plus defoliation(Li et al., 2015a).

Gene name	TPM value						log2(FoldChange)			P Value		
	CK2	CK4	CK7	GPD2	GPD4	GPD7	CK2-vs-GPD 2	CK4-vs-GPD 4	CK7-vs-GPD 7	CK2-vs-GPD 2	CK4-vs-GPD 4	CK7-vs-GPD 7
LcCCD1	58.99	57.46	54.59	30.08	40.22	55.79	-0.93	-0.36	0.12	0.00	0.00	0.23
LcCCDlike-a	0.00	0.00	0.00	0.00	0.00	0.00	0.00	0.00	0.00	1.00	1.00	1.00
LcCCDlike-b	0.00	1.36	0.00	0.00	1.35	0.64	0.00	0.15	9.43	1.00	1.00	1.00
LcCCD4	68.51	113.00	87.66	82.77	98.20	144.83	0.32	-0.04	0.82	0.01	0.75	0.00
LcCCD4a1	0.00	0.00	0.74	0.18	0.00	0.00	8.37	0.00	-10.37	1.00	1.00	0.50
LcCCD4a2	1.14	1.53	2.22	0.10	3.86	0.91	-3.42	1.48	-1.20	0.22	0.08	0.34
LcCCD4b	0.38	0.00	0.59	0.00	1.09	0.90	-9.41	11.08	0.70	0.50	0.25	1.00
LcCCD4c1	0.00	0.54	0.58	0.00	0.81	0.77	0.00	0.74	0.49	1.00	1.00	1.00
LcCCD4c2	0.22	0.00	0.00	0.00	0.00	0.73	-8.61	0.00	10.43	1.00	1.00	0.50
LcCCD7	0.00	0.29	0.00	0.00	0.00	0.00	0.00	0.00	0.00	1.00	1.00	1.00
LcCCD8a	21.86	14.92	10.00	0.00	34.39	0.00	-15.28	1.36	-14.18	0.00	0.00	0.00
LcCCD8b	0.20	1.83	0.25	0.25	2.74	0.00	0.42	0.74	-8.94	1.00	0.45	1.00
LcNCED1	40.95	11.12	34.42	15.28	13.76	9.78	-1.38	0.46	-1.73	0.00	0.19	0.00
LcNCED2	27.74	88.35	33.42	66.88	78.49	33.39	1.34	0.00	0.08	0.00	1.00	0.66
LcNCED3	1.85	4.49	9.06	2.90	5.27	4.35	0.71	0.39	-0.97	0.38	0.61	0.04

Supplement Table S10. The TPM value and differential expression analysis of *LcCCO* genes of abscission zone samples of 'Feizixiao'litchi after 0, 1, 2, and 3 days treated by exogenous ethephon (Li et al., 2015b).

Gene name	TPM value							log2(FoldChange)						P Value					
	CK0	CK1	CK2	CK3	ETH1	ETH2	ETH3	CK0-vs-CK1	CK0-vs-CK2	CK0-vs-CK3	CK0-vs-ETH1	CK0-vs-ETH2	CK0-vs-ETH3	CK0-vs-CK1	CK0-vs-CK2	CK0-vs-CK3	CK0-vs-ETH1	CK0-vs-ETH2	CK0-vs-ETH3
LcCCD1	91.57	89.52	88.08	76.00	21.01	35.43	65.06	0.02	-0.05	-0.19	-2.12	-1.34	-0.38	0.68	0.31	0.00	0.00	0.00	0.00
LcCCDlike-a	0.25	0.00	0.29	0.47	0.44	0.47	0.00	-2.34	0.19	0.97	0.80	0.92	-2.34	0.22	1.00	0.42	0.42	0.42	0.22
LcCCDlike-b	0.00	0.91	0.82	0.57	0.36	0.38	0.70	2.36	2.16	1.69	0.96	1.08	2.04	0.22	0.38	0.63	1.00	1.00	0.38
LcCCD4	17.86	35.47	33.35	18.69	6.99	8.37	103.29	1.05	0.95	0.14	-1.33	-1.02	2.64	0.00	0.00	0.33	0.00	0.00	0.00
LcCCD4a1	0.15	0.00	0.18	0.19	0.12	0.20	0.14	-0.50	0.32	0.46	-0.19	0.50	0.12	1.00	1.00	1.00	1.00	1.00	1.00
LcCCD4a2	3.99	3.86	2.15	3.40	0.37	0.32	0.57	0.00	-0.88	-0.16	-3.43	-3.58	-2.71	1.00	0.03	0.72	0.00	0.00	0.00
LcCCD4b	0.98	1.66	1.16	1.14	0.60	0.72	0.35	0.81	0.25	0.28	-0.72	-0.41	-1.37	0.25	0.82	0.82	0.45	0.80	0.18
LcCCD4c1	0.22	0.65	0.00	0.00	0.00	0.45	0.00	1.61	0.16	0.08	0.00	1.08	0.00	0.63	1.00	1.00	1.00	1.00	1.00
LcCCD4c2	0.10	0.72	0.34	0.75	0.41	0.11	0.30	2.84	1.75	2.92	1.98	0.08	1.61	0.07	0.63	0.07	0.38	1.00	0.63
LcCCD7	0.00	0.00	0.00	0.00	0.00	0.00	0.00	0.00	0.16	0.08	0.00	0.08	0.00	1.00	1.00	1.00	1.00	1.00	1.00
LcCCD8a	76.21	86.43	72.64	66.91	0.00	12.40	22.41	0.24	-0.03	-0.11	-9.63	-2.56	-1.66	0.00	0.71	0.17	0.00	0.00	0.00
LcCCD8b	2.34	2.19	2.61	1.65	0.09	0.36	0.51	-0.04	0.20	-0.42	-4.71	-2.65	-2.09	1.00	0.78	0.45	0.00	0.00	0.00
LcNCED1	93.07	32.92	49.74	40.32	12.10	14.22	46.64	-1.44	-0.88	-1.13	-2.94	-2.67	-0.89	0.00	0.00	0.00	0.00	0.00	0.00
LcNCED2	238.83	238.95	244.37	251.53	483.58	339.90	399.29	0.07	0.08	0.17	1.05	0.59	0.86	0.11	0.05	0.00	0.00	0.00	0.00
LcNCED3	0.90	0.61	0.58	0.83	0.09	0.45	0.34	-0.49	-0.58	-0.04	-3.33	-0.94	-1.29	0.63	0.45	1.00	0.01	0.30	0.18

Supplement Table S11. The TPM value and differential expression analysis of *LcCCO* genes of the peel samples on 0d and 4d after stored at room tempera ture and 0h, 24h, and 48h stored at room temperature after precooling for 14 days (Yun et al., 2016).

Gene name	TPM value					log2(FoldChange)						P Value					
	0 d	4 d	14 d-0 h	14 d-24 h	14 d-48 h	0 d-vs-4d	0 d-vs-14 d-0 h	0 d-vs-14 d-24 h	0 d-vs-14 d-48 h	14 d-0 h-vs-14 d-24 h	14 d-0 h-vs-14 d-48 h	0 d-vs-4d	0 d-vs-14 d-0 h	0 d-vs-14 d-24 h	0 d-vs-14 d-48 h	14 d-0 h-vs-14 d-24 h	14 d-0 h-vs-14 d-48 h
LcCCD1	17.67	10.17	9.55	20.83	12.01	-0.56	-0.80	0.17	-0.50	0.96	0.30	0.00	0.00	0.00	0.00	0.00	0.00
LcCCDlike-a	0.21	0.22	2.23	0.29	0.29	0.30	3.50	0.41	0.52	-3.10	-2.99	0.58	0.00	0.51	0.34	0.00	0.00
LcCCDlike-b	0.37	0.12	0.16	0.16	0	-1.45	-1.19	-1.27	-3.04	-0.08	-1.85	0.34	0.34	0.23	0.04	1.00	0.63
LcCCD4	21.22	7.06	7.29	17.61	14.35	-1.27	-1.42	-0.31	-0.41	1.11	1.01	0.00	0.00	0.00	0.00	0.00	0.00
LcCCD4a1	0.05	0	10.88	0.17	0.11	-0.32	7.83	1.63	1.14	-6.20	-6.69	1.00	0.00	0.29	0.69	0.00	0.00
LcCCD4a2	0.03	0	4.73	0.08	0	0.68	7.63	1.68	0.00	-5.95	-7.63	1.00	0.00	0.63	1.00	0.00	0.00
LcCCD4b	0	0	0.83	0	0	0.68	5.11	0.26	0.00	-4.85	-5.11	1.00	0.00	1.00	1.00	0.00	0.00
LcCCD4c1	0	0	0	0	0	0.68	0.26	0.26	0.00	0.00	-0.26	1.00	1.00	1.00	1.00	1.00	1.00
LcCCD4c2	0	0	0	0	0	0.68	0.26	0.26	0.00	0.00	-0.26	1.00	1.00	1.00	1.00	1.00	1.00
LcCCD7	0	0.06	0	0	0	1.58	0.26	0.26	0.00	0.00	-0.26	1.00	1.00	1.00	1.00	1.00	1.00
LcCCD8a	0	0.05	0.04	0	0	1.14	0.85	0.26	0.00	-0.58	-0.85	1.00	1.00	1.00	1.00	1.00	1.00
LcCCD8b	0.03	0.03	0.11	0.03	0.02	0.68	2.26	0.26	0.00	-2.00	-2.26	1.00	0.38	1.00	1.00	0.38	0.38
LcNCED1	94.26	40.49	17.07	128.54	61.02	-0.97	-2.37	0.38	-0.55	2.75	1.82	0.00	0.00	0.00	0.00	0.00	0.00
LcNCED2	51.34	10.42	6.91	21.48	30.1	-2.03	-2.78	-1.32	-0.66	1.46	2.12	0.00	0.00	0.00	0.00	0.00	0.00
LcNCED3	0.03	0.06	0.08	0	0.05	1.58	1.85	0.26	1.14	-1.58	-0.71	1.00	0.63	1.00	1.00	0.63	1.00
